# Fast random opposition-based learning Aquila optimization algorithm

**DOI:** 10.1016/j.heliyon.2024.e26187

**Published:** 2024-02-15

**Authors:** S. Gopi, Prabhujit Mohapatra

**Affiliations:** Department of Mathematics, School of Advanced Sciences, Vellore Institute of Technology, Vellore, 632 014, Tamil Nadu, India

**Keywords:** Opposition-based learning, Optimization algorithms, Meta-heuristic algorithm, Fast random opposition-based learning, OBL, FROBL

## Abstract

Meta-heuristic algorithms are usually employed to address a variety of challenging optimization problems. In recent years, there has been a continuous effort to develop new and efficient meta-heuristic algorithms. The Aquila Optimization (AO) algorithm is a newly established swarm-based method that mimics the hunting strategy of Aquila birds in nature. However, in complex optimization problems, the AO has shown a sluggish convergence rate and gets stuck in the local optimal region throughout the optimization process. To overcome this problem, in this study, a new mechanism named Fast Random Opposition-Based Learning (FROBL) is combined with the AO algorithm to improve the optimization process. The proposed approach is called the FROBLAO algorithm. To validate the performance of the FROBLAO algorithm, the CEC 2005, CEC 2019, and CEC 2020 test functions, along with six real-life engineering optimization problems, are tested. Moreover, statistical analyses such as the Wilcoxon rank-sum test, the t-test, and the Friedman test are performed to analyze the significant difference between the proposed algorithm FROBLAO and other algorithms. The results demonstrate that FROBLAO achieved outstanding performance and effectiveness in solving an extensive variety of optimization problems.

## 1 Introduction

Optimization is the act of determining the supreme combination of decision factors to address a certain optimization problem. This approach has surfaced in a variety of fields, restraints, and real-life applications [1], [2], [3]. Finding answers to optimization problems has become the standard in almost all fields of engineering and sciences [4], [5], [6], where the desire for more powerful solutions is always growing. This implies that we must have sensible algorithms capable of dealing with the complexities of present-day scientific and engineering problems. An extensive review of the literature on existing meta-heuristic algorithms reveals that there are numerous such methods [7], [8], [9]. These methods range from traditional techniques that employ either linear or non-linear programming methods [10] to newly developed, nature-inspired methods, each with its own set of advantages and disadvantages. Traditional approaches, while effective in tackling well-known optimization issues [11], [12], [13], have two drawbacks: they require a full-promise initial start vector within the search area, and they are inherently dependent on gradient information [14], [15]. Also, traditional approaches can no longer be employed to address challenging real-world problems as an outcome of the improvement of technology and science in high society. Consequently, the meta-heuristic algorithm was developed. Because of their straightforward design and adaptable parameters, meta-heuristic algorithms are frequently applied to these challenging real-world problems [16]. Due to their benefits, including flexibility, efficiency, and the ability to produce a solution that is close to optimal in a fair amount of time, meta-heuristic algorithms have been utilized to address such problems. Meta-heuristic algorithms mimic unpredictability in nature to find the optimum solution. These algorithms can be separated into four classes: evolutionary algorithms, physics-based algorithms, swarm-based algorithms, and human-based algorithms. (i) Evolutionary algorithms: this is the most frequent and earliest type of meta-heuristic algorithm, imitating the principles of evolutionary behavior of species in nature by depending on the idea of continuity of the qualified. In evolutionary algorithms, an initial random population improves throughout iterations to create new solutions and eliminate the poorest ones to improve the fitness value. Given that they lack credibility in the basic fitness landscape, these algorithms frequently do well in discovering optimal or near-optimal solutions. (ii) Physics-based algorithms: these algorithms take their inspiration from the establishing physical fundamentals prevalent throughout the universe. These algorithms evolved from natural physics laws and typically separated the interaction of search agents based on the existing principles of physical processes. (iii) Swarm-based algorithms: this is an important branch of meta-heuristics that approaches the dynamic, collective, intelligent, and concerted gregarious behavior of flocks in nature. The communities mentioned above consist of flocks of birds, fish schooling, insect colonies such as bee and ant colonies, flocks of animals, and numerous flocks of other species of organisms. Each character in swarm-based algorithms has its own wit and behavior, but combining characters in algorithms provides additional power to tackle complicated optimization problems. (iv) Human-based algorithms: this group characterizes phenomena connected to human behavior, non-physical activities like thinking, and their perceptions in society. The algorithms in this class have begun to grab the attention of researchers as a new trend in the last decade and are still unable to compete with evolutionary and swarm-based algorithms. Table 1 illustrates the meta-heuristic algorithms categorization.

**Table 1:** Categorization of meta-heuristic algorithms.

Category	Algorithm
Evolutionary algorithms	Evolution Strategy (ES) [17]
	Genetic Algorithm (GA) [18]
	Biogeography-Based Optimizer (BBO) [19]
	Genetic Programming (GP) [20]
	Differential Evolution (DE) [21]
	Evolutionary Deduction Algorithm (ED) [22]
	Probability-Based Incremental Learning (PBIL) [23]
	Tree Growth Algorithm (TGA) [24]
	Arithmetic Optimization Algorithm (AOA) [25]

Physics-based algorithms	Gravitational Local Search (GLSA) [26]
	Gravitational Search Algorithm (GSA) [27]
	Simulated Annealing (SA) [28]
	Multi-verse Optimizer (MVO) [29]
	Electromagnetic Field Optimization (EFO) [30]
	Equilibrium Optimizer (EO) [31]
	Central Force Optimization (CFO) [32]
	Big-Bang Big-Crunch (BBBC) [33]
	Ray Optimization (RO) [34]
	Henry Gas Solubility Optimization (HGSO) [35]
	Curved Space Optimization (CSO) [36]

Swarm-based algorithms	Ant Colony Optimization (ACO) [37]
	Salp Swarm Algorithm (SSA) [38]
	Particle Swarm Optimization (PSO) [39], [40]
	Artificial Bee Colony (ABC) [41]
	Ant Lion Optimizer (ALO) [42]
	Slime Mold Algorithm (SMA) [43]
	Cuckoo Search (CS) [44]
	Moth Flame Optimization (MFO) [45]
	Firefly Algorithm(FA) [46]
	Seagull optimization algorithm (SOA) [47]
	Grey Wolf Optimizer (GWO) [48]
	American zebra optimization algorithm (AZOA) [49]
	Sooty Tern Optimization Algorithm (STOA) [50]
	Whale Optimization Algorithm (WOA) [51]
	Bald Eagle Search (BES) [52]
	Marine Predators Algorithm (MPA) [53]

Human-based algorithms	Collective Decision Optimization (CSO) [54]
	Teaching–learning-based optimization (TLBO) [55]
	Harmony Search (HS) [56]
	Fireworks Algorithm (FWA) [57]
	Socio Evolution & Learning Optimization Algorithm (SELOA) [58]
	Poor and Rich Optimization (PRO) [59]
	Human Eye Vision Algorithm (HEVA) [60]
	Brain Storm Optimization Algorithm (BSOA) [61]
	Human Mental Search (HMS) [62]

The recently developed algorithm, the Aquila optimizer (AO), which imitates the four different stages of Aquila hunting behavior, was proposed by Abualigah et al. [14]. By swapping the AO's original exploitation phase with the Harris Hawks Optimizer's exploitation phase, Wang et al. [63] created an improved version of the AO. They also included a nonlinear escape operator and a random opposition-learning technique in their proposed algorithm. Furthermore, the AO and the arithmetic optimization algorithm (AOA) [25] were hybridized by Mahajan et al. [64], named the hybrid algorithm of arithmetic optimization algorithm with AO algorithm (AOAAO). Then the AOAAO algorithm results are compared with the original AO, original AOA, Grey Wolf algorithm (GWO), Grasshopper algorithm (GOA) [65], and Whale algorithm (WOA). The simplified AO method was created by Zhao et al. [66] by maintaining the first two techniques and eliminating the control equation from the exploitation and exploration processes. They employed 23 functions to evaluate their implemented approach using different optimizers. Furthermore, Gao et al. [67], implemented three different methods to improve the AO algorithm. These techniques involve developing a search control operator, random-opposition learning, and Gaussian mutation (GM). They claimed that their method, an improved AO, provided better results compared to those of other optimizers. Furthermore, Huangjing et al. [68], engaged 3 different approaches to enhance the AO algorithm. These approaches are the restart strategy, opposition-based learning, and chaotic local search. Also, Yufei Wang et al. [69], introduced a new strategy, called an adaptive opposition-based learning strategy. This strategy improves local optima for the AO algorithm. Furthermore, Ekinci et al. [70], introduced a novel strategy, namely an enhanced AO (enAO) algorithm by employing two mechanisms such as the Nelder-Mead (NM) method and the modified opposition-based learning (OBL) strategy to improve the exploration and exploitation. The AO has been applied successfully in a variety of applications. AlRassas et al. [71], for instance, attempted to forecast oil production by utilizing the AO to maximize the model of an adaptive neuro-fuzzy inference system. Despite the algorithm's strength and superiority, as well as corresponding to the NFL theorem [72], the AO can't solve every optimization problem. Therefore, the AO still requires improvements and innovations.

This paper introduces a new strategy, namely the Fast Random Opposition-Based Learning (FROBL) strategy [73], and this provides a novel contribution with the AO algorithm, Fast Random Opposition-Based Learning Aquila Optimizer (FROBLAO), to reformulate the update customs to prevent falling into local optima while escalating convergence. The use of FROBL in collaboration with the optimization functions of AO raises the certainty and performance of the natural AO approximately. The implementation's benefits involve helping the algorithm depart the local optimal while still maintaining complexity and AO's optimization progress. Therefore, it is hoped that the proposed FROBLAO algorithm's adaptability will be demonstrated by careful testing later in the paper.

The following are the main features of this paper:

• A advanced AO algorithm has been developed using the Fast Random Opposition-Based Learning strategy, namely the FROBLAO algorithm.
• The proposed FROBLAO algorithm has been compared with the original AO, opposition-based learning with AO, namely OBLAO, five popular algorithms, two top-performing algorithms, and two recent high-performance algorithms, such as AO [14], OBLAO, TSA [74], SSA [38], MVO [29], GWO [48], SCA [75], LSHADE [76], CMA-ES [77], MRFO [78] and AVOA [79].
• FROBLAO was tested for CEC 2005, CEC 2019, and CEC 2020 test functions, along with six engineering design problems.

The remaining part of this study is formulated as follows: Section 2 discusses the preliminaries of the original AO algorithm, OBL strategy, FROBL strategy, and proposed FROBLAO algorithm. Section 3 discusses the experimental results and discussions for the test functions. Section 4 discusses the real-life engineering problems. At last section 5 discusses the conclusion of the proposed work.

## 2 Preliminaries

### 2.1 Aquila optimizer (AO)

One of the most recent optimizer for population-based swarm intelligence is the Aquila Optimization algorithm. One of the most well-known predatory birds that once inhabited the northern hemisphere was the Aquila. The body and back of the Aquila are golden. Aquila catches a variety of prey, primarily squirrels, rabbits, marmots, and hares, using her quickness and strength, as well as its strong feet and wide claws. The AO simulates the four various hunting techniques and those techniques are modeled mathematically as follows:

*Phase 1: expanded exploration (*$X_{1}$*)*  The Aquila soars high above ground level during this phase to properly scan the area before diving vertically after it has discovered the prey. This behavior is expressed in two mathematical equations as follows:

(1) $$X_{1}(l+1)=X_{B}(l)⁎(1-\frac{l}{L})+(X_{mean}(l)-X_{B}(l)⁎rand)$$

(2) $$X_{mean}(l)=\frac{1}{M}\sum_{i=1}^{M}X_{i}(l),\forall M=1,2,\cdots ,Dim$$

where $X_{mean}(l)$ denotes the mean location of the present solutions at $i^{th}$ iteration by using Equation (2), $X_{B}$ is the global best solution in this iteration, rand stands for random values that lies in [0,1], *l* stands for the current iteration, *L* signifies the number of iteration, *M* express the population size, *Dim* express the dimension size.

*Phase 2: narrowed exploration (*$X_{2}$*)*  The majority of Aquila's hunting methods involve this particular phase. Contour flying is combined with a brief glide to attack the prey. The following Equations are updates to Aquila positions:

(3) $$X_{2}(l+1)=X_{B}(l)⁎Levy(D)+X_{R}(l)+(y-x)⁎rand$$

where $X_{R}$ express the random location of the Aquila, *D* denotes the dimension space, Levy denotes the levy probability distribution function which is evaluated by employing Equations (4), (5), (6), and (7).

(4) $$Levy(D)=s⁎\frac{u⁎\sigma }{|\nu {|}^{\frac{1}{\beta }}}$$

(5) $$\sigma =\frac{\Gamma (1+\beta )⁎sin(\frac{\pi \beta }{2})}{\Gamma (\frac{1+\beta }{2})⁎\beta ⁎2^{\frac{\beta -1}{2}}}$$

Here, *s* and *β* denote the constant whose values are 0.01 and 1.5, respectively. The *u* and *ν* are arbitrary numbers lies in 0 and 1. The following two equations can be used to calculate the values of *y* and *x*, which are employed to simulate the spiral shape:

(6) y=γ⁎cos(θ)

(7) x=γ⁎sin(θ)

where *γ* and *θ* can be determined by using Equations (8), (9), and (10).

(8) $$\gamma =\gamma_{1}+V⁎D_{1}$$

(9) $$\theta =-W⁎D_{1}+\theta_{1}$$

(10) $$\theta_{1}=\frac{3⁎\pi }{2}$$

where $\gamma_{1}$ takes a value between 1 and 20, *V* is equal to 0.0265, *W* is equal to 0.005, and $D_{1}$ denotes the random integer from the range one to the dimension.

*Phase 3: expanded exploitation (*$X_{3}$*)*  The third phase involves locating the prey location so that agents can launch a low-flying preliminary strike vertically. The following are some possible ways that agents can attack their prey:

(11) $$X_{3}(l+1)=(X_{B}(l)-X_{mean}(l))⁎\alpha -rand+((\mathrm{UB}-\mathrm{LB})⁎rand+\mathrm{LB})⁎\delta$$

where *α* and *δ* are exploitation fixed parameters to 0.1, and UB and LB denotes the upper and lower limits.

*Phase 4: narrowed exploitation (*$X_{4}$*)*  The fourth phase involves the Aquila's ability to quickly track and attack its target using escape trajectory light, which is calculated using Equation (12), (13), (14), and (15).

(12) $$X_{4}(l+1)=QF⁎X_{B}(l)-(P_{1}⁎X(l)⁎rand)-P_{2}⁎Levy(D)+rand⁎P_{1}$$

(13) $$QF(l)=l^{\frac{2⁎rand-1}{{\left(1-\gamma \right)}^{2}}}$$

(14) $$P_{1}=2⁎rand-1$$

(15) $$P_{2}=2⁎(1-\frac{l}{L})$$

where QF(l) denotes the quality value, $P_{1}$ denotes the various motions of AO, and $P_{2}$ denotes the chasing target flight slope.

### 2.2 Opposition-based learning (OBL)

Opposition-Based Learning (OBL) is one of the effective optimization tools that was developed by Tizhoosh in 2005 [80]. The fundamental idea of the OBL method is to simultaneously evaluate the fitness of an estimate and the opposite estimate that corresponds to it, to find a superior candidate solution. Several meta-heuristic algorithms have effectively employed the OBL principle to speed up convergence. The definition of the OBL strategy, let consider $\overset{ˆ}{a}$ is the opposite value for the real value *a* ∈ [LB, UB], which is calculated using Equation (16).

(16) $$\overset{ˆ}{a}=\mathrm{LB}+\mathrm{UB}-a.$$

**Figure 1:**
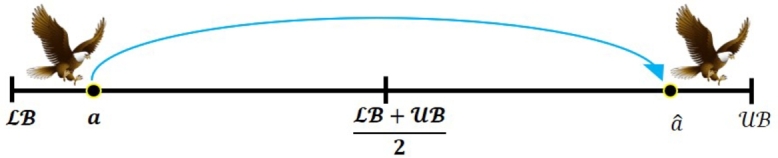
One dimensional space of OBL strategy.

**Figure 2:**
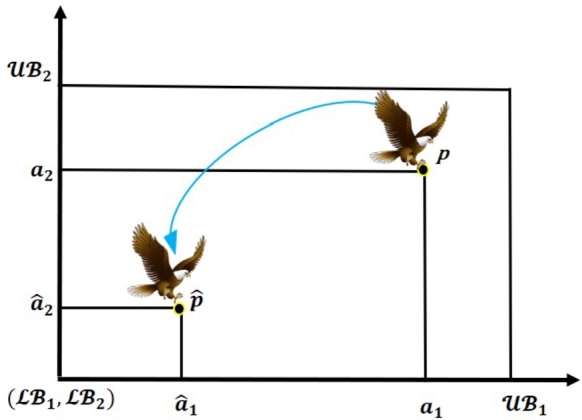
Two dimensional space of OBL strategy.

**Figure 3:**
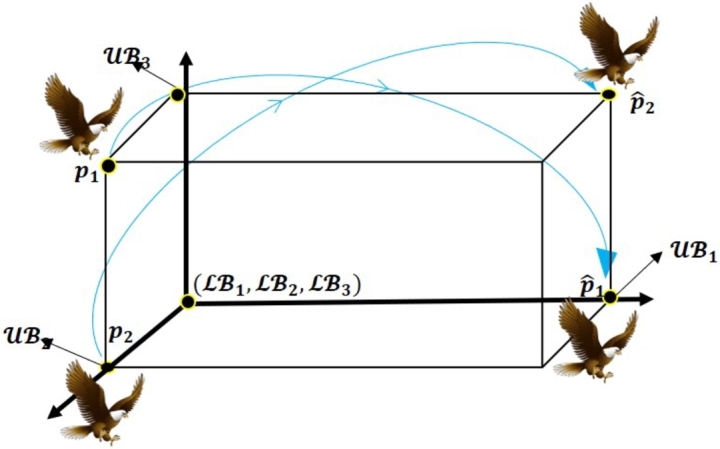
Three dimensional space of OBL strategy.

The following equation can be employed to expand this definition to n dimensions:

(17) $${\overset{ˆ}{a}}_{i}={\mathrm{LB}}_{i}+{\mathrm{UB}}_{i}-a_{i},$$

where the ${\overset{ˆ}{a}}_{i}$ and *a* are two solutions related during the optimization process; the superior of these solutions is maintained, while the other is eliminated through the evaluation of the objective function, for example, if $f(\overset{ˆ}{a})$ ≤ f(a) (if minimum), then *a* is maintained; otherwise $\overset{ˆ}{a}$ is maintained. Fig. 1, Fig. 2, and Fig. 3 denote the formation of *a* and its opposite $\overset{ˆ}{a}$ in one, two, and three-dimensional spaces, respectively.

### 2.3 Proposed method

#### 2.3.1 Fast random opposition-based learning (FROBL)

In 2023, Sarada et al. [73] successfully developed the novel Fast Random Opposition-Based Learning (FROBL) technique to increase the diversity (exploration) and intensification (exploitation) abilities of the algorithm. This innovative FROBL approach is enforced to basic solutions to achieve a unique solution that constructs adverse populations to enhance the diversity and assist the population to jump out from the local optima. Assume that the opposite value $\overset{ˆ}{a}$ for the real value *a* ∈ [LB, UB], which is evaluated by employing Equation (18).

(18) $$\overset{ˆ}{a}=\left\{ \begin{array}{c} M+r^{2}⁎sin(2⁎\pi ⁎r)⁎\frac{a}{2},||a||<||M||;\\ M-r^{2}⁎sin(2⁎\pi ⁎r)⁎\frac{a}{2},otherwise. \end{array}\right.$$

**Figure 4:**
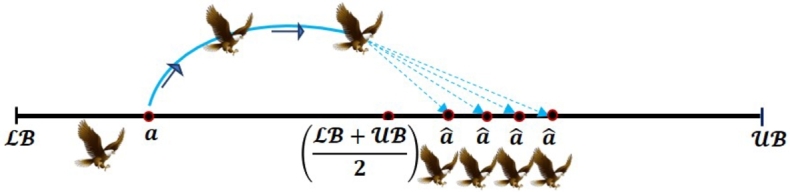
One dimensional space of FROBL strategy.

**Figure 5:**
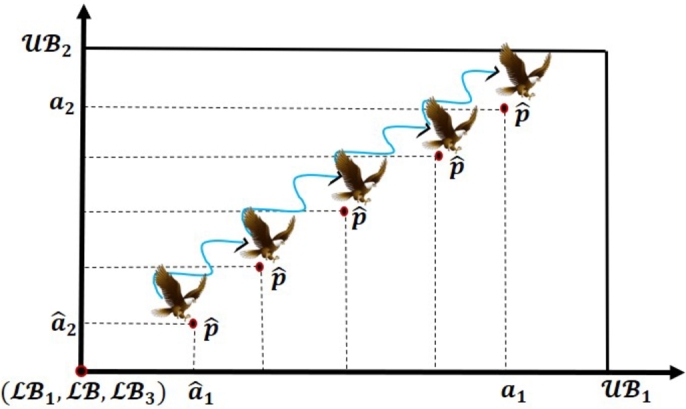
Two dimensional space of FROBL strategy.

**Figure 6:**
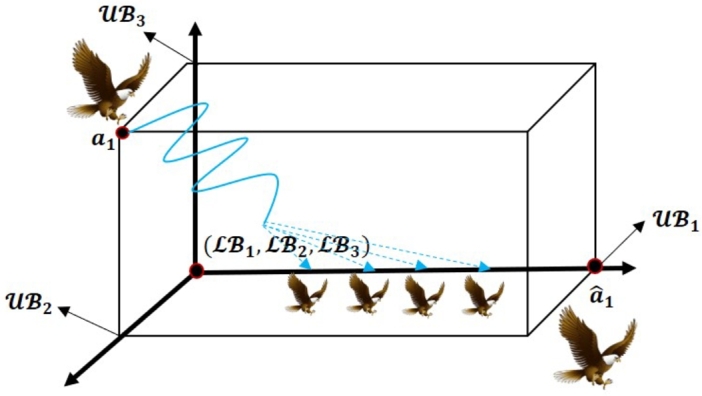
Three dimensional space of FROBL strategy.

The following equation can be employed to expand this definition to n dimensions:

(19) $${\overset{ˆ}{a}}_{i,j}=\left\{ \begin{array}{c} M+r^{2}⁎sin(2⁎\pi ⁎r)⁎\frac{a_{i,j}}{2},||a_{i,j}||<||M||;\\ M-r^{2}⁎sin(2⁎\pi ⁎r)⁎\frac{a_{i,j}}{2},otherwise, \end{array}\right.$$

where $M=\frac{\mathrm{LB}+\mathrm{UB}}{2}$ and *r* express the random value lies between 0 and 1, and *π* value is 3.14. The |||| denotes the Euclidean distance between the origin to the position of the particle. The sine function represents the cyclic structure combined with *π* and *r* according to the solution can be relocated around another solution. This can ensure that the region defined between the two solutions is exploited. The solutions should be able to search outside the distance between their corresponding destinations to fully explore the search space. The new functions have been introduced to modify the update conditions in comparison to Equation (17) to prevent increased convergence while also avoiding poor diversity and local optima. Fig. 4, Fig. 5, and Fig. 6 express the formation of *a* and its opposite $\overset{ˆ}{a}$ in one, two, and three-dimensional spaces, respectively.

#### 2.3.2 Fast random opposition-based learning Aquila optimizer (FROBLAO)

**Figure 7:**
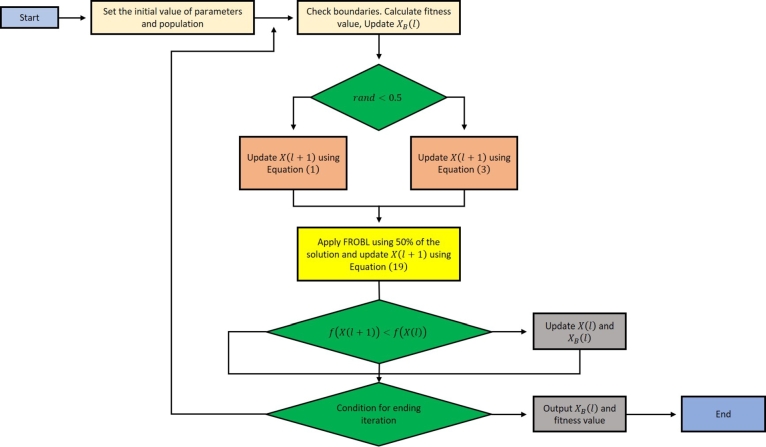
The flowchart of the FROBLAO algorithm.

For improving the performance of the AO algorithm, the structure of the proposed method is described in this section. To introduce the capability of thoroughly exploring the search domain and quickly reaching the optimal value, the AO is modified in this context by combining its original structure with the FROBL strategy. After the exploration phase of the AO is complete, the FROBL technique is used to maintain 25% of the AO-calculated domain space. This process permits the initial domain space to quickly approach the optimum value and restore the out-of-range values. The proposed method has been named FROBLAO, and Algorithm 1 illustrates its main steps. According to the NFL (No Free Lunch) theorem, no optimization solution can effectively address every optimization problem. According to the NFL, no algorithm can be improved without sacrificing benefits. The proposed FROBLAO algorithm pseudocode is shown in Algorithm 1, and Fig. 7 depicts the flowchart of the FROBLAO algorithm.

**Algorithm 1:**
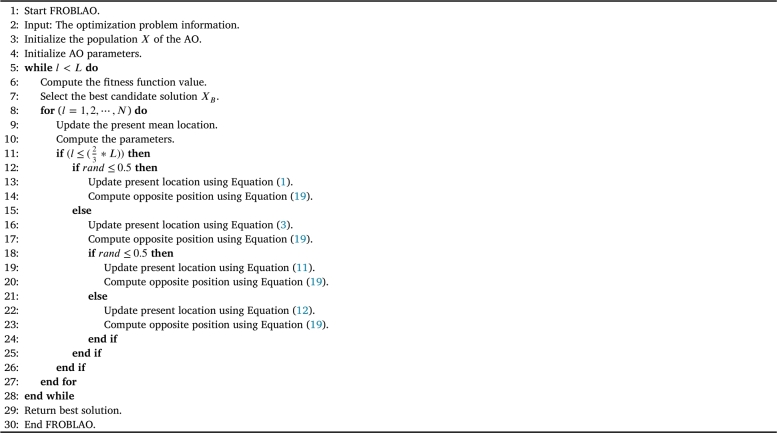
The proposed FROBLAO's pseudo-code.

## 3 Experimental results and discussions

This study is conducted to analyze the FROBLAO algorithm's performance by employing a variety of test functions. In the numeric process of authentication, forty-three collections of benchmark test functions have been used, namely twenty-three CEC 2005 benchmark functions [81], [82], ten CEC 2019 benchmark functions [83] and ten CEC 2020 benchmark functions [84]. The details of the CEC 2005, CEC2019, and CEC2020 test functions are given in Table 2, Table 3, and Table 4, respectively. The FROBLAO algorithm has been run thirty times independently to evaluate its stability and dependability. The average (Mean) value and standard deviation (Std) value of the FROBLAO algorithm have been provided to compare with AO variants, popular algorithms, top-performing algorithms, and recent high-performance algorithms, such as AO [14], OBLAO, TSA [74], SSA [38], MVO [29], GWO [48], SCA [75], LSHADE [76], CMA-ES [77], MRFO [78], and AVOA [79]. All the algorithm's initial parameters are given in the Table 5. To make a valid comparison, the algorithms under evaluation have used the same number of runs, maximum number of population sizes, maximum number of iterations of 30, 30, and 500, respectively. All the experiments are carried out on Windows 11, Intel Core i3, 2.10 GHz, 8.00 GB RAM, MABLAB R2022b.

**Table 2:** CEC 2005 test functions.

Function	Dim	Range	*F*_*min*_
$F1(h)=\sum_{i=1}^{n}{h_{i}}^{2}$	30	[−100,100]	0
$F2\left(h\right)=\sum_{i=1}^{n}|h|+\;\prod_{i}^{n}|h_{i\;}|$	30	[−10,10]	0
$F3\left(h\right)=\sum_{i=1}^{n}{\left(\sum_{j=1}^{i}h_{j}\right)}^{2}$	30	[−100,100]	0
$F4\left(h\right)=max_{i}\left\{|h_{i}|,\;\;1\leq i\leq n\right\}$	30	[−100,100]	0
$F5\left(h\right)=\sum_{i=1}^{n-1}[100{\left(h_{i+1}-h_{i}^{2}\right)}^{2}+{\left(h_{i}-1\right)}^{2}]$	30	[−30,30]	0
$F6\left(h\right)=\sum_{i=1}^{n}{\left([h_{i}+0.5]\right)}^{2}$	30	[−100,100]	0
$F7\left(h\right)=\;\sum_{i=1}^{n}ih_{i}^{4}+random[0,1)$	30	[−1.28,1.28]	0
$F8(h)=\sum_{i=1}^{n}-h_{i}\sin(\sqrt{|h_{i}}|)$	30	[−500,500]	−12569.5
$F9\left(h\right)=\sum_{i=1}^{n}[h_{i}^{2}-10\cos(2\pi h_{i})+10]$	30	[−5.12,5.12]	0
$F10\left(h\right)=-20exp(-0.2\sqrt{\frac{1}{n}}\sum_{i=1}^{n}h_{i}^{2})-exp(\frac{1}{n}\sum_{i=1}^{n}\cos(2\pi h_{i}))+20+e$	30	[−32,32]	0
$F11\left(h\right)=\frac{1}{4000}\sum_{i=1}^{n}h_{i}^{2}-\prod_{i=1}^{n}\cos(\frac{h_{i}}{\sqrt{i}})+1$	30	[−600,600]	0
$F12\left(h\right)=\frac{\pi }{n}\left\{10\sin\pi y_{1})+\sum_{i=1}^{n-1}{\left(y_{i}-1\right)}^{2}\left[1+10sin^{2}\left(\pi y_{i+1}\right)\right]+{\left(y_{n}-1\right)}^{2}\right\}+\sum_{i=1}^{n}u(h_{i},10,100,4)$	30	[−50,50]	0
$y_{i}=1+\frac{h_{i}+1}{4}$			
$u(h_{i},a,k,m)=\left\{ \begin{array}{cc} k{\left(h_{i}-a\right)}^{m}\; & \;h_{i}>a\\ 0\; & -a<h_{i}<a\\ k{\left(-h_{i}-a\right)}^{m}\; & h_{i}\;<-a \end{array}\right.$			
$F13\left(h\right)=0.1\{(3\pi h_{1})+\sum_{i=1}^{n}{\left(h_{i}-1\right)}^{2}[1+sin^{2}(3\pi h_{i}+1)]+{\left(h_{n}-1\right)}^{2}[1+sin^{2}(2\pi h_{n}]\}+\sum_{i=1}^{n}u(h_{i},5,100,4)$	30	[−50,50]	0
$F14\left(h\right)={\left(\frac{1}{500}+\sum_{j=1}^{25}\frac{1}{j+\sum_{i=1}^{2}{\left(h_{i}-a_{ij}\right)}^{6}}\right)}^{-1}$	2	[−65,65]	1
$F15\left(h\right)=\sum_{i=1}^{11}{\left[a_{i}-\frac{h_{1}(b_{i}^{2}+b_{i}h_{2})}{b_{i}^{2}+b_{i}h_{3}+h_{4}}\right]}^{2}$	4	[−5,5]	0.0003
$F16\left(h\right)=4h_{1}^{2}-2.1h_{1}^{4}+\frac{1}{3}h_{1}^{6}+\;h_{1}h_{2}-4h_{2}^{2}+4h_{2}^{4}$	2	[−5,5]	−1.0316
$F17\left(h\right)={\left(h_{2}-\frac{5.1}{4\pi^{2}}h_{1}^{2}+\frac{5}{\pi }h_{1}-6\right)}^{2}+10(1-\frac{1}{8\pi })\cos h_{1}+10$	2	[−5,5]	0.398
$F18\left(h\right)=[1+{\left(h_{1}+h_{2}+1\right)}^{2}(19-14h_{1}+3h_{1}^{2}-14h_{2}+6h_{1}h_{2}+3h_{2}^{2})]\times [30+{\left(2h_{1}-3h_{2}\right)}^{2}(18-32h_{1}+12h_{1}^{2}+48h_{2}-36h_{1}h_{2}+27h_{2}^{2})]$	2	[−2,2]	3
$F19\left(h\right)=-\sum_{i=1}^{4}c_{i}exp(-\sum_{j=1}^{3}a_{ij}{\left(h_{j}-p_{ij}\right)}^{2})$	3	[1,3]	−3.86
$F20\left(h\right)=-\sum_{i=1}^{4}c_{i}exp(-\sum_{j=1}^{6}a_{ij}{\left(h_{j}-p_{ij}\right)}^{2})$	6	[0,1]	−3.32
$F21\left(h\right)=-\sum_{i=1}^{5}{\left[(H-a_{i}){\left(H-a_{i}\right)}^{T}+c_{i}\right]}^{-1}$	4	[0,10]	−10.1532
$F22\left(h\right)=-\sum_{i=1}^{7}{\left[(H-a_{i}){\left(H-a_{i}\right)}^{T}+c_{i}\right]}^{-1}$	4	[0,10]	−10.4028
$F23\left(h\right)=-\sum_{i=1}^{10}{\left[(H-a_{i}){\left(H-a_{i}\right)}^{T}+c_{i}\right]}^{-1}$	4	[0,10]	−10.5363

**Table 3:** CEC 2019 test functions.

Functions	Dim	Range	*CEC*_*min*_
*CEC*01 (*Storn*′*s chebyshev polynomial fitting problem*)	9	[−8192,8192]	1
*CEC*02 (*Inverse Hilbert matrix problem*)	16	[−16384,16384]	1
*CEC*03 (*Lennard* − *jones minimum energy cluster*)	18	[−4,4]	1
*CEC*04 (*Rastrigin*′*s function*)	10	[−100,100]	1
*CEC*05 (*Griewangk*′*s function*)	10	[−100,100]	1
*CEC*06 (*Weierstrass function*)	10	[−100,100]	1
*CEC*07 (*Modified schwefel*′*s function*)	10	[−100,100]	1
*CEC*08 (*Expanded Schaffer*′*s F*6 *function*)	10	[−100,100]	1
*CEC*09 (*Happy cat function*)	10	[−100,100]	1
*CEC*10 (*Ackley function*)	10	[−100,100]	1

**Table 4:** CEC 2020 test functions.

Functions	Dim	Range	*H*_*min*_
*H*1 (*CEC*2017,*F*1)(*Shifted and Rotated Bent Cigar Function*)	10	[−100,100]	100
*H*2 (*CEC*2014,*F*11)(*Shifted and Rotated Schwefel*′*s Function*)	10	[−100,100]	1100
*H*3 (*CEC*2017,*F*7)(*Shifted and Rotated Lunacek bi* − *Rastrigin Function*)	10	[−100,100]	700
*H*4 (*CEC*2017,*F*19)(*Expanded Rosen brock*′*s plus Griewangk*′*s Function*)	10	[−100,100]	1900
*H*5 (*CEC*2014,*F*17)(*Hybrid Function* 1)	10	[−100,100]	1700
*H*6 (*CEC*2017,*F*16)(*Hybrid Function* 2)	10	[−100,100]	1600
*H*7 (*CEC*2014,*F*21)(*Hybrid Function* 3)	10	[−100,100]	2100
*H*8 (*CEC*2017,*F*22)(*Composition Function* 1)	10	[−100,100]	2200
*H*9 (*CEC*2017,*F*24)(*Composition Function* 2)	10	[−100,100]	2400
*H*10 (*CEC*2017,*F*25)(*Composition Function* 3)	10	[−100,100]	2500

**Table 5:** The control parameters of the competing algorithms are set to certain values.

Algorithm	Parameter	Value
*FROBLAO*	*V*, *γ*_1_, *W*, *α*, *δ*	0.0265, 10, 0.005, 0.1, 0.1
*AO*	*V*, *γ*_1_, *W*, *α*, *δ*	0.0265, 10, 0.005, 0.1, 0.1
*OBLAO*	*V*, *γ*_1_, *W*, *α*, *δ*	0.0265, 10, 0.005, 0.1, 0.1
*TSA*	*P*_*min*_, *P*_*max*_	1, 4
*SSA*	*Leader position update probability*	0.5
*MVO*	*WEP*_*Max*, *WEP*_*Min*	1, 0.2
*GWO*	*l*, *r*	[−1,1], [0,1]
*SCA*	*a*	2
*LSHADE*	*Pbest rate*,*Arc rate*,*Memory size*	0.11, 1.4, 5
*CMAES*	*α*	2
*MRFO*	*S*, *r*_1_, *r*_2_, *r*_3_	2, [0,1], [0,1], [0,1]
*AVOA*	*α*, *β*, *δ*, *P*_1_, *P*_2_, *P*_3_	0.8, 0.2, 2.5, [0,1], [0,1], [0,1]

### 3.1 Time complexity of the FROBLAO

By evaluating the time complexity of the initialization, calculation, and updating position processes independently, it is possible to determine the time complexity of the proposed FROBLAO algorithm. Therefore, O(FROBLAO) = *O*(Initialize the position) + *O*(Calculate the fitness value) + *O*(Update the position) + O(FROBL). Assume that *M* express the number of populations, *L* express the total number of iterations, and *Dim* represents the number of dimensions. Then the time complexity of updating the proposed FROBLAO is described as follows:

*O*(Initialize the position) = O(M)

*O*(Calculate the fitness value) = O(M×L)

*O*(Update the position) = O(M×L×Dim)

O(FROBL) = O(M×L×Dim).

Hence the total time complexity of the proposed FROBLAO is equal to O(FROBLAO) = O(M) + O(M×L) + O(M×L×Dim) + O(M×L×Dim) = O(M×L×Dim).

### 3.2 Measurements of performance

• **Average (**Mean**):** The average value can be calculated using Equation (20).
  (20) $$Mean=\frac{1}{N}\sum_{i=1}^{N}M_{i},$$
  where, $M_{i}$ signifies the better solution accomplished from $i^{th}$ run and *N* denotes 30 independent runs.
• **Standard deviation (***Std***):** For calculating the standard deviation, use Equation (21).
  (21) $$Std=\sqrt{\frac{1}{N}\sum_{i=1}^{N}{\left(M_{i}-M\right)}^{2}},$$
  where, *M* denotes the mean of the 30 runs, $M_{i}$ signifies the better solution accomplished from $i^{th}$ run and *N* denotes 30 independent runs.
• **t-test:** A statistical test, such as a t-test, is used to determine the significant differences between the proposed method and other meta-heuristics. These are calculated using Equation (22).
  (22) $$t-cost=\frac{Mean_{1}-Mean_{2}}{\sqrt{\frac{Std_{1}^{2}+Std_{2}^{2}}{N}}},$$
  where, $Mean_{1}$, $Mean_{2}$, $Std_{1}$, and $Std_{2}$ be the average and std for the two different algorithms.

### 3.3 Comparison results of CEC 2005 test functions

In this subsection, we discuss the efficiency of the proposed FROBLAO and compare it with AO variants, popular algorithms, and top-performing algorithms. Table 6 demonstrates the statistical outcomes of competitive algorithms, like mean and standard deviation results. In comparison to AO, FROBLAO produces better results in 20 functions (*F*1 to *F*7, and *F*12 to *F*23), whereas FROBLAO produces the same results in 3 functions (*F*9 to *F*11). In comparison to OBLAO, FROBLAO produces better results in 13 functions (*F*5 to *F*7, *F*12 to *F*18, and *F*20 to *F*22), whereas FROBLAO produces the same results in 9 functions (*F*1 to *F*4, *F*8 to *F*11, and *F*19). In comparison to TSA, FROBLAO produces better results in 22 functions (*F*1 to *F*7, and *F*9 to *F*23). In comparison to SSA, FROBLAO produces better results in 23 functions. Results were similar when compared to GWO and SCA algorithms. In comparison to MVO, FROBLAO produces better results in 22 functions (*F*1 to *F*13 and *F*15 to *F*23). In comparison to LSHADE, FROBLAO produces better results in 19 functions (*F*1 to *F*7, *F*9 to *F*13, *F*15, and *F*18 to *F*23), whereas FROBLAO produces the same results in 3 functions (*F*14, *F*16, and *F*17). In comparison to CMA-ES, FROBLAO produces better results in 19 functions (*F*1 to *F*7, *F*9 to *F*15, *F*18, and *F*20 to *F*23), whereas FROBLAO produces the same results in 3 functions (*F*16, *F*17, and *F*19). In comparison to MRFO, FROBLAO produces better results in 11 functions (*F*2, *F*4, *F*5, *F*7, *F*12, *F*13, *F*15, and *F*20 to *F*23), whereas FROBLAO produces the same results in 9 functions (*F*1, *F*3, *F*9 to *F*11, *F*14, *F*16 to *F*18). In comparison to AVOA, FROBLAO produces better results in 18 functions (*F*1 to *F*7, *F*12 to *F*15, and *F*17 to *F*23), whereas FROBLAO produces the same results in 4 functions (*F*9 to *F*11 and *F*16). Hence, the proposed FROBLAO algorithm has a good exploitation ability and a good spatial exploration ability, which makes it possible for it to handle optimization problems successfully.

**Table 6:** Results of CEC 2005 test functions on FROBLAO to compare with other algorithms.

Functions	*AO*	*OBLAO*	*TSA*	*SSA*	*MVO*	*GWO*	*SCA*	*LSHADE*	*CMA* − *ES*	*MRFO*	*AVOA*	*FROBLAO*
**F1**												
*Mean*	1.7163E-111	**0**	2.5252E-21	1.7682E-07	1.4701E-02	2.4998E-27	6.7704E-12	1.0820E-01	1.1333E-05	**0**	5.9672E-302	**0**
*Std*	9.4008E-111	**0**	6.5609E-21	2.1148E-07	7.5624E-03	4.8313E-27	2.6459E-11	1.4983E-01	4.6012E-06	**0**	0	**0**

**F2**												
*Mean*	3.8906E-55	**0**	7.8971E-14	1.3435E-02	3.7292E-02	8.5561E-17	1.1659E-09	1.4620E-01	5.1791E-03	1.2923E-205	1.0365E-148	**0**
*Std*	2.1293E-54	**0**	8.0448E-14	3.7141E-02	1.3708E-02	5.5446E-17	2.6378E-09	1.6020E-01	1.5425E-03	0	5.6753E-148	**0**

**F3**												
*Mean*	5.7030E-102	**0**	3.1817E-04	7.7395E-07	1.2050E-01	8.7098E-05	1.2070E-02	5.1011E+02	4.3656E-01	**0**	1.1742E-228	**0**
*Std*	3.1220E-101	**0**	7.4468E-04	2.2226E-06	7.3420E-02	2.5361E-04	4.8676E-02	3.2016E+02	2.1998E-01	**0**	0	**0**

**F4**												
*Mean*	1.2953E-51	**0**	2.9942E-01	4.2172E-05	8.4682E-02	8.1885E-07	2.3103E-03	1.4483E+01	2.3320E-02	5.5620E-200	4.7178E-145	**0**
*Std*	7.0907E-51	**0**	3.5015E-01	1.1192E-04	2.3954E-02	1.1604E-06	4.9523E-03	3.0416E+00	6.2271E-03	0	2.5798E-144	**0**

**F5**												
*Mean*	1.9136E-03	1.0983E-05	2.8236E+01	1.6916E+02	2.5696E+02	2.7139E+01	7.4362E+00	1.5630E+02	4.3648E+01	2.2849E+01	4.6278E-05	**2.8763E-07**
*Std*	2.5562E-03	9.3549E-06	9.5719E-01	4.8774E+02	5.1973E+02	8.2774E-01	4.1879E-01	1.1556E+02	4.3488E+01	4.7244E-01	3.6732E-05	**3.5913E-07**

**F6**												
*Mean*	2.0674E-05	1.0051E-06	3.5443E+00	8.8172E-10	1.4239E-02	7.4424E-01	4.3737E-01	5.1390E-02	1.0834E-05	**0**	4.7940E-07	1.2535E-08
*Std*	2.9670E-05	1.0187E-06	7.3726E-01	3.3196E-10	6.4076E-03	3.6459E-01	1.7695E-01	4.5090E-02	4.0063E-06	**0**	2.7300E-07	2.1314E-08

**F7**												
*Mean*	9.1969E-05	1.0125E-05	1.0861E-02	1.5511E-02	3.1080E-03	1.8978E-03	2.4682E-03	8.4200E-02	2.4237E-02	1.5070E-04	1.5228E-04	**4.3932E-07**
*Std*	1.1608E-04	5.9546E-06	5.7467E-03	8.9977E-03	2.3910E-03	9.8852E-04	1.7364E-03	3.9555E-02	6.4990E-03	1.4087E-04	1.3585E-04	**9.4442E-07**

**F8**												
*Mean*	-3.6295E+03	-4.1898E+03	-5.8489E+03	-2.6808E+03	-2.9450E+03	-5.9188E+03	-2.2056E+03	-1.2117E+04	**-1.1770E+05**	-8.2331E+03	-1.2195E+04	-4.1898E+03
*Std*	8.0617E+02	1.1158E-02	6.4755E+02	3.1134E+02	3.2022E+02	1.0274E+03	1.7423E+02	1.9635E+02	**6.5578E+04**	7.5921E+02	9.1477E+02	1.3412E-02

**F9**												
*Mean*	**0**	**0**	1.8217E+02	1.5090E+01	1.4599E+01	4.0971E+00	1.5276E+00	5.4683E+00	1.3110E+02	**0**	**0**	**0**
*Std*	**0**	**0**	4.6500E+01	7.1178E+00	5.8972E+00	4.4188E+00	7.0681E+00	2.5698E+00	7.0854E+01	**0**	**0**	**0**

**F10**												
*Mean*	**4.4409E-16**	**4.4409E-16**	1.6831E+00	7.3684E-01	5.8901E-01	1.0430E-13	1.0500E-03	2.9171E+00	1.0363E-03	**4.4409E-16**	**4.4409E-16**	**4.4409E-16**
*Std*	**3.0088E-31**	**3.0088E-31**	1.6232E+00	9.8093E-01	6.4644E-01	1.9608E-14	5.7332E-03	8.3701E-01	2.4638E-04	**3.0088E-31**	**3.0088E-31**	**3.0088E-31**

**F11**												
*Mean*	**0**	**0**	1.1127E-02	1.9688E-01	3.6576E-01	3.1189E-03	7.6846E-02	1.5184E-01	1.3608E-04	**0**	**0**	**0**
*Std*	**0**	**0**	1.0123E-02	1.0612E-01	1.3126E-01	7.2341E-03	1.0683E-01	1.2428E-01	4.5562E-05	**0**	**0**	**0**

**F12**												
*Mean*	9.5407E-06	2.1123E-07	7.1429E+00	7.4179E-01	3.2364E-02	4.9874E-02	9.2963E-02	1.6222E+00	1.3504E-06	9.1020E-09	2.2530E-08	**5.8385E-12**
*Std*	2.3033E-05	1.6230E-07	3.2399E+00	1.0493E+00	9.6468E-02	2.2068E-02	3.2778E-02	1.4785E+00	6.0598E-07	1.9949E-08	1.3650E-08	**1.3199E-11**

**F13**												
*Mean*	1.7980E-05	1.4196E-07	3.0684E+00	1.7995E-03	6.5473E-03	5.1398E-01	3.1885E-01	3.0158E+00	1.5074E-05	2.4219E+00	4.6219E-08	**1.9155E-08**
*Std*	4.2318E-05	1.1463E-07	6.1632E-01	4.9376E-03	5.3018E-03	1.8370E-01	1.0016E-01	3.7334E+00	5.8219E-06	1.1242E+00	3.3144E-08	**3.1011E-08**

**F14**												
*Mean*	2.5053E+00	1.3948E+00	9.8551E+00	1.2297E+00	**9.9800E-01**	4.6626E+00	1.8907E+00	**9.9800E-01**	4.9447E+00	**9.9800E-01**	1.4280E+00	**9.9800E-01**
*Std*	2.7753E+00	8.0721E-01	4.7878E+00	5.6408E-01	**3.3876E-16**	3.6583E+00	1.8950E+00	**3.3876E-16**	3.0422E+00	**3.3876E-16**	8.1025E-01	**3.3876E-16**

**F15**												
*Mean*	4.9413E-04	3.3059E-04	1.2708E-02	4.0687E-03	4.2868E-03	5.7283E-03	1.0920E-03	4.4194E-04	2.3521E-03	4.4973E-04	3.6969E-04	**3.1016E-04**
*Std*	1.0819E-04	1.6409E-05	2.6755E-02	7.4170E-03	7.4084E-03	8.9776E-03	3.7198E-04	3.5372E-04	1.3403E-03	3.5189E-04	1.0432E-04	**2.8158E-06**

**F16**												
*Mean*	-1.0312E+00	-1.0316E+00	-1.0316E+00	-1.0316E+00	-1.0316E+00	-1.0316E+00	-1.0316E+00	**-1.0316E+00**	**-1.0316E+00**	**-1.0316E+00**	**-1.0316E+00**	**-1.0316E+00**
*Std*	4.2135E-04	1.0501E-05	3.9165E-07	2.3469E-14	4.0256E-07	2.8019E-08	7.6526E-05	**0**	**0**	**0**	**0**	**0**

**F17**												
*Mean*	3.9804E-01	3.9789E-01	3.9794E-01	3.9789E-01	3.9789E-01	3.9790E-01	4.0016E-01	**3.9789E-01**	**3.9789E-01**	**3.9789E-01**	3.9789E-01	**3.9790E-01**
*Std*	2.4679E-04	7.4273E-06	5.3285E-05	1.3357E-14	8.2593E-07	6.3724E-05	2.2952E-03	**1.1292E-16**	**1.1292E-16**	**1.1292E-16**	8.7029E-16	**1.1292E-16**

**F18**												
*Mean*	3.0285E+00	3.0015E+00	1.1368E+01	3.0000E+00	5.7000E+00	3.0000E+00	3.0001E+00	3.0000E+00	3.0000E+00	**3.0000E+00**	3.0000E+00	**3.0000E+00**
*Std*	2.7722E-02	1.4704E-03	2.2418E+01	1.8192E-13	1.4789E+01	5.4917E-05	1.8330E-04	2.5657E-15	2.5657E-15	**1.8142E-15**	9.1421E-06	**1.8142E-15**

**F19**												
*Mean*	-3.8551E+00	-3.8625E+00	-3.8624E+00	-3.8628E+00	-3.8628E+00	-3.8608E+00	-3.8548E+00	-3.0048E-01	**-3.8628E+00**	**-3.8628E+00**	-3.8628E+00	-3.8625E+00
*Std*	5.3641E-03	2.7689E-04	1.4292E-03	7.1564E-11	1.5117E-06	2.9353E-03	3.1724E-03	0	**2.7101E-15**	**2.7101E-15**	2.3705E-11	2.6409E-04

**F20**												
*Mean*	-3.1293E+00	-3.2306E+00	-3.2517E+00	-3.2270E+00	-3.2493E+00	-3.2496E+00	-2.9377E+00	-3.3022E+00	-3.2705E+00	-3.2863E+00	-3.2859E+00	**-3.3197E+00**
*Std*	8.9217E-02	6.1566E-02	7.3541E-02	5.9148E-02	6.0363E-02	1.1545E-01	3.3429E-01	4.5066E-02	5.9923E-02	5.5415E-02	5.6017E-02	**2.2248E-03**

**F21**												
*Mean*	-1.0129E+01	-1.0153E+01	-5.2940E+00	-7.3946E+00	-6.9634E+00	-8.3909E+00	-2.6755E+00	-8.6519E+00	-8.4084E+00	-8.7937E+00	-1.0153E+01	**-1.0153E+01**
*Std*	5.1031E-02	4.6493E-04	2.9851E+00	3.3091E+00	3.1573E+00	2.8084E+00	2.2941E+00	2.8296E+00	3.2168E+00	2.2930E+00	5.4758E-05	**7.4626E-13**

**F22**												
*Mean*	-1.0393E+01	-1.0403E+01	-6.4279E+00	-8.8387E+00	-8.3044E+00	-1.0401E+01	-3.4356E+00	-9.6205E+00	-1.0403E+01	-8.6312E+00	-1.0403E+01	**-1.0403E+01**
*Std*	1.4369E-02	4.6314E-04	3.5541E+00	2.9226E+00	2.8591E+00	9.6699E-04	1.9653E+00	2.0646E+00	0	2.5485E+00	4.0616E-05	**1.0137E-13**

**F23**												
*Mean*	-1.0524E+01	-1.0536E+01	-6.5208E+00	-8.6442E+00	-8.3275E+00	-1.0535E+01	-3.7448E+00	-1.0134E+01	-1.0266E+01	-9.4548E+00	-1.0536E+01	**-1.0536E+01**
*Std*	2.3294E-02	3.5260E-04	3.7865E+00	3.2224E+00	3.2621E+00	1.2180E-03	1.6062E+00	1.5402E+00	1.4815E+00	2.2002E+00	1.0265E-03	**2.4788E-13**

### 3.4 Comparison results of CEC 2019 test functions

In this subsection, we discuss the efficiency of the proposed FROBLAO and compare it with AO variants, popular algorithms, top-performing algorithms, and recent high-performance algorithms. Table 7 demonstrates the statistical outcomes of competitive algorithms, like mean and standard deviation results. In comparison to AO, FROBLAO produces better results in 10 functions. Results were similar when compared to the OBLAO, TSA, SSA, GWO, SCA, and AVOA algorithms. In comparison to MVO, FROBLAO produces better results in 9 functions (CEC01 to CEC03 and CEC05 to CEC10). In comparison to LSHADE, FROBLAO produces better results in 7 functions (CEC01, CEC02, and CEC06 to CEC10). Results were similar when compared to the MRFO algorithm. In comparison to CMA-ES, FROBLAO produces better results in 8 functions (CEC01 to CEC03 and CEC06 to CEC10). Hence, the proposed FROBLAO algorithm has a good exploitation ability and a good spatial exploration ability, which makes it possible for it to handle optimization problems successfully.

**Table 7:** Results of CEC 2019 test functions on FROBLAO to compare with other algorithms.

Functions	*AO*	*OBLAO*	*TSA*	*SSA*	*MVO*	*GWO*	*SCA*	*LSHADE*	*CMA* − *ES*	*MRFO*	*AVOA*	*FROBLAO*
**CEC01**												
*Mean*	5.8162E+04	4.3500E+04	4.1623E+08	6.9628E+09	4.0931E+09	2.6073E+08	8.3745E+09	2.0417E+07	3.8659E+08	3.8362E+04	4.6338E+04	**3.7674E+04**
*Std*	1.2167E+04	3.1767E+03	8.4702E+08	8.1850E+09	2.9698E+09	5.2126E+08	1.1427E+10	5.3397E+07	2.9751E+08	7.1216E+02	4.2265E+03	**9.3083E+02**

**CEC02**												
*Mean*	1.7371E+01	1.7349E+01	1.8309E+01	1.7347E+01	1.8954E+01	1.7344E+01	1.7480E+01	1.7343E+01	1.0647E+02	1.7347E+01	1.7343E+01	**1.7343E+01**
*Std*	1.2233E-02	2.5097E-03	5.6451E-01	1.3780E-02	7.9543E-01	3.1615E-04	8.1389E-02	3.4407E-14	4.6060E+01	7.8493E-04	7.4226E-08	**0**

**CEC03**												
*Mean*	1.2702E+01	1.2702E+01	1.2703E+01	1.2702E+01	1.2702E+01	1.2702E+01	1.2703E+01	**1.2702E+01**	1.2702E+01	**1.2702E+01**	1.2702E+01	**1.2702E+01**
*Std*	6.3450E-06	4.1688E-07	1.4095E-03	2.6513E-11	4.6965E-09	3.6996E-06	1.2184E-04	**9.0336E-15**	1.3329E-04	**9.0336E-15**	1.9787E-09	**9.0336E-15**

**CEC04**												
*Mean*	6.6005E+02	5.0794E+01	4.2087E+03	4.7559E+01	3.8414E+01	2.0358E+02	1.7648E+03	**9.5887E+00**	2.1930E+01	3.3299E+01	1.3232E+02	4.3987E+01
*Std*	5.4468E+02	2.1377E+01	2.7740E+03	2.7312E+01	1.2130E+01	6.5305E+02	9.8911E+02	**4.2700E+00**	6.9889E+00	1.9416E+01	6.1470E+01	2.0421E+01

**CEC05**												
*Mean*	1.5377E+00	1.2566E+00	2.6898E+00	1.2305E+00	1.3061E+00	1.4244E+00	2.2222E+00	1.0357E+00	**1.0276E+00**	1.1439E+00	1.4281E+00	1.2104E+00
*Std*	1.7008E-01	1.3845E-01	5.8219E-01	1.2223E-01	1.4195E-01	2.6916E-01	1.0522E-01	2.6004E-02	**3.4842E-02**	1.0126E-01	3.2860E-01	9.0954E-02

**CEC06**												
*Mean*	1.0662E+01	8.3942E+00	1.1122E+01	6.2934E+00	8.4414E+00	1.0866E+01	1.0838E+01	8.7360E+00	1.0517E+01	1.0426E+01	6.3738E+00	**5.7330E+00**
*Std*	1.1467E+00	9.8413E-01	6.3773E-01	3.5783E-01	1.1857E+00	5.8197E-01	6.7443E-01	1.9273E+00	6.5943E-01	7.2000E-01	1.5924E+00	**9.0350E-01**

**CEC07**												
*Mean*	3.6239E+02	1.4045E+02	6.5854E+02	3.0983E+02	3.1114E+02	4.8478E+02	8.3450E+02	2.4262E+02	2.8153E+02	2.3616E+02	3.6043E+02	**3.3918E+01**
*Std*	1.6835E+02	1.3710E+02	2.2301E+02	2.2817E+02	2.2466E+02	3.1424E+02	1.6636E+02	1.5391E+02	2.4113E+02	1.3423E+02	1.9949E+02	**1.1933E+02**

**CEC08**												
*Mean*	5.4593E+00	4.5671E+00	6.2935E+00	5.0889E+00	5.3875E+00	5.0082E+00	6.0604E+00	4.5729E+00	5.1209E+00	4.6699E+00	5.7072E+00	**3.3854E+00**
*Std*	7.1525E-01	5.5741E-01	5.7205E-01	6.1588E-01	5.8004E-01	1.1802E+00	4.1349E-01	5.3735E-01	1.7053E+00	8.9526E-01	5.0759E-01	**7.6536E-01**

**CEC09**												
*Mean*	5.1682E+00	2.4782E+00	4.7013E+02	2.6484E+00	2.4781E+00	4.4617E+00	1.2319E+02	2.4700E+00	2.4009E+00	2.3690E+00	3.3665E+00	**2.3572E+00**
*Std*	8.5265E-01	8.3635E-02	5.4262E+02	1.5708E-01	6.5192E-02	7.8120E-01	1.0049E+02	1.1041E-01	1.2125E-02	2.0034E-02	5.0230E-01	**9.3767E-03**

**CEC10**												
*Mean*	1.8980E+01	1.1731E+01	2.0504E+01	2.0038E+01	2.0104E+01	2.0472E+01	2.0508E+01	2.0098E+01	2.0433E+01	1.6432E+01	2.0045E+01	**4.6705E+00**
*Std*	4.4574E+00	9.6746E+00	7.6757E-02	6.4503E-02	4.5829E-02	2.3391E-01	1.0464E-01	8.5590E-02	8.3900E-02	8.0856E+00	4.8026E-02	**7.8510E+00**

### 3.5 Comparison results of CEC 2020 test functions

In this subsection, we discuss the efficiency of the proposed FROBLAO and compare it with AO variants, popular algorithms, top-performing algorithms, and recent high-performance algorithms. Table 8 demonstrates the statistical outcomes of competitive algorithms, like mean and standard deviation results. In comparison to AO, FROBLAO produces better results in 10 functions. Results were similar when compared to the OBLAO, TSA, GWO, and SCA algorithms. In comparison to SSA, FROBLAO produces better results in 9 functions (*H*2 to *H*10). In comparison to MVO, FROBLAO produces better results in 8 functions (*H*2 to *H*9). In comparison to LSHADE, FROBLAO produces better results in 8 functions (*H*2 and *H*4 to *H*10). In comparison to CMA-ES, FROBLAO produces better results in 9 functions (*H*1 to *H*7, *H*9, and *H*10). In comparison to MRFO, FROBLAO produces better results in 7 functions (*H*2 to *H*7 and *H*9). In comparison to AVOA, FROBLAO produces better results in 7 functions (*H*2 to *H*8). Hence, the proposed FROBLAO algorithm has a good exploitation ability and a good spatial exploration ability, which makes it possible for it to handle optimization problems successfully.

**Table 8:** Results of CEC 2020 test functions on FROBLAO to compare with other algorithms.

Functions	*AO*	*OBLAO*	*TSA*	*SSA*	*MVO*	*GWO*	*SCA*	*LSHADE*	*CMA* − *ES*	*MRFO*	*AVOA*	*FROBLAO*
**H1**												
*Mean*	5.3937E+07	6.6997E+04	3.7038E+09	9.5186E+03	1.8114E+04	4.1726E+07	1.1156E+09	**1.0178E+02**	1.9557E+06	2.2555E+03	1.3503E+03	3.6926E+04
*Std*	1.2885E+08	3.6281E+04	3.2885E+09	1.4634E+04	6.3596E+03	1.2054E+08	3.5526E+08	**5.4331E+00**	2.3868E+06	2.2992E+03	6.7700E+02	1.7903E+04

**H2**												
*Mean*	1.8928E+03	1.6988E+03	2.2212E+03	2.0386E+03	1.8327E+03	1.6993E+03	2.6044E+03	1.5674E+03	2.7081E+03	1.9045E+03	2.0143E+03	**1.4400E+03**
*Std*	2.5834E+02	2.4273E+02	2.4395E+02	4.5386E+02	3.6731E+02	3.3469E+02	1.9383E+02	1.4373E+02	2.8746E+02	3.0315E+02	3.0895E+02	**1.2494E+02**

**H3**												
*Mean*	7.5764E+02	7.4425E+02	7.9804E+02	7.3953E+02	7.3142E+02	7.3475E+02	7.8508E+02	**7.1924E+02**	7.4131E+02	7.4716E+02	7.7124E+02	7.2467E+02
*Std*	1.3519E+01	1.3226E+01	2.6156E+01	1.6429E+01	1.1723E+01	1.0591E+01	1.0617E+01	**2.5758E+00**	5.3341E+00	1.7168E+01	2.7312E+01	9.1039E+00

**H4**												
*Mean*	2.6933E+04	2.9383E+03	2.2371E+05	2.8999E+04	4.7528E+03	1.0046E+04	1.1533E+04	1.9023E+03	1.0481E+04	2.0784E+03	3.5674E+03	**1.9011E+03**
*Std*	3.9796E+04	2.0497E+03	4.3595E+05	3.7706E+04	4.6306E+03	7.8146E+03	6.8273E+03	6.5134E-01	9.5934E+03	1.7093E+02	2.4832E+03	**0**

**H5**												
*Mean*	2.3973E+04	1.0122E+04	4.3749E+05	2.7461E+04	5.3430E+03	6.0681E+04	7.4164E+04	2.1038E+03	2.2704E+05	4.8614E+03	2.9098E+04	**1.9249E+03**
*Std*	4.6236E+04	1.9864E+03	3.7701E+05	4.0720E+04	3.3606E+03	1.4449E+05	8.4941E+04	1.4989E+02	3.0841E+05	2.6132E+03	3.3555E+04	**5.9604E+01**

**H6**												
*Mean*	1.8223E+03	1.6758E+03	1.9372E+03	1.8425E+03	1.8010E+03	1.7269E+03	1.8190E+03	1.6190E+03	1.6944E+03	1.7180E+03	1.6895E+03	**1.6030E+03**
*Std*	1.4345E+02	1.0138E+02	1.1380E+02	1.3211E+02	1.4509E+02	9.8699E+01	1.0883E+02	3.1654E+01	7.3012E+01	1.3524E+02	7.4358E+01	**4.9858E+00**

**H7**												
*Mean*	1.7860E+04	6.4242E+03	3.6986E+04	1.0420E+04	9.1449E+03	9.5589E+03	1.5709E+04	2.1363E+03	1.8374E+04	2.5611E+03	8.6528E+03	**2.1005E+03**
*Std*	1.3407E+04	3.6964E+03	6.6871E+04	1.0591E+04	6.8934E+03	5.8879E+03	7.9336E+03	4.6843E+01	1.8243E+04	3.9366E+02	7.6166E+03	**4.0189E-02**

**H8**												
*Mean*	2.3108E+03	2.3031E+03	2.5290E+03	2.3412E+03	2.3291E+03	2.3131E+03	2.3954E+03	2.3000E+03	2.3000E+03	**2.2995E+03**	2.3500E+03	2.3009E+03
*Std*	1.3011E+01	1.0623E+01	2.3526E+02	2.0494E+02	1.3665E+02	9.4496E+00	4.9442E+01	2.9714E-06	5.9561E-10	**1.3422E+01**	0	1.7646E+00

**H9**												
*Mean*	2.7495E+03	2.7289E+03	2.8375E+03	2.7491E+03	2.7325E+03	2.7547E+03	2.7912E+03	2.7626E+03	2.7549E+03	2.7017E+03	**2.6000E+03**	2.6308E+03
*Std*	8.0106E+01	7.8274E+01	3.5210E+01	6.9673E+01	6.3609E+01	1.3915E+01	8.5907E+00	9.2825E+01	9.5704E+00	1.0332E+02	**0**	6.2973E+01

**H10**												
*Mean*	2.9318E+03	2.7289E+03	3.0582E+03	2.9412E+03	2.9156E+03	2.9367E+03	2.9787E+03	2.9304E+03	2.9312E+03	2.9137E+03	**2.7000E+03**	2.9266E+03
*Std*	2.4452E+01	7.8274E+01	1.4756E+02	3.3831E+01	2.2960E+01	1.6963E+01	2.2485E+01	2.1707E+01	2.1181E+01	6.3589E+01	**0**	2.3041E+01

### 3.6 Statistical analysis

The minimum mean value is superior to the algorithm's ability to discover a solution nearly as good as the optimum solution. Although the average values of the two algorithms may be equivalent, their performance in achieving the optimal solution may differ in every iteration. However, Std has been employed to provide a more appropriate comparison. The standard deviation should be small to have a smaller range of results. Statistical tests are necessary to fully evaluate the performance of the meta-heuristic algorithms. It is insufficient to compare meta-heuristic algorithms based on their average and standard deviation values; a statistical test is required to demonstrate a significant improvement of a new meta-heuristic algorithm to overtake the other meta-heuristic algorithms for solving particular optimization problems. The assumption of normality is simply the presumption that a random variable of interest, or in our instance, the data from the data sample, follows the normal probability distribution bu using average and standard deviation. A Kolmogorov-Smirnov test is used to determine normality [85], while Levene's test, which is based on averages, is used to determine homoscedasticity [86]. Histograms and quantile-quantile plots, or Q-Q plots, are two other graphical methods for determining the normality of the data [87]. The *NA* denotes “Not Applicable” which means that the FROBLAO algorithm results are similar to the compared algorithm results.

• **Wilcoxon rank-sum:** Moreover, Table 9 represents the Wilcoxon rank-sum test [88] results on CEC 2005 test functions, Table 10 represents the Wilcoxon rank-sum test results on CEC 2019 test functions, and Table 11 represents the Wilcoxon rank-sum test results on CEC 2020 test functions, which are used to compare the proposed FROBLAO algorithm's statistical performance to that of other algorithms. It is important to remember that a p−cost of less than 0.05 denotes a significant difference between the two compared algorithms. The FROBLAO superiors all other algorithms to varied degrees regarding this condition. The symbols + express that FROBLAO is better, = expresses that FROBLAO is identical, and − expresses that FROBLAO is poorer than the comparable algorithm. *Hyp* denotes the hypothesis. In conclusion, the FROBLAO algorithm outperforms other comparison algorithms on a majority of test functions.

**Table 9:** Results on Wilcoxon rank-sum test for FROBLAO with other compared algorithms of CEC 2005 test functions.

Functions	AO vs FROBLAO		OBLAO vs FROBLAO		TSA vs FROBLAO		SSA vs FROBLAO		MVO vs FROBLAO		GWO vs FROBLAO	
	*p* − *cost*	*Hyp*	*p* − *cost*	*Hyp*	*p* − *cost*	*Hyp*	*p* − *cost*	*Hyp*	*p* − *cost*	*Hyp*	*p* − *cost*	*Hyp*
**F1**	1.6783*E* − 06	+	*NA*	=	1.6783*E* − 06	+	1.6783*E* − 06	+	1.6783*E* − 06	+	1.6783*E* − 06	+
**F2**	1.6783*E* − 06	+	*NA*	=	1.6783*E* − 06	+	1.6783*E* − 06	+	1.6783*E* − 06	+	1.6783*E* − 06	+
**F3**	1.6783*E* − 06	+	*NA*	=	1.6783*E* − 06	+	1.6783*E* − 06	+	1.6783*E* − 06	+	1.6783*E* − 06	+
**F4**	1.6783*E* − 06	+	*NA*	=	1.6783*E* − 06	+	1.6783*E* − 06	+	1.6783*E* − 06	+	1.6783*E* − 06	+
**F5**	1.6783*E* − 06	+	1.7571*E* − 06	+	1.6783*E* − 06	+	1.6783*E* − 06	+	1.6783*E* − 06	+	1.6783*E* − 06	+
**F6**	1.6783*E* − 06	+	2.5802*E* − 06	+	1.6783*E* − 06	+	5.8925*E* − 01	−	1.6783*E* − 06	+	1.6783*E* − 06	+
**F7**	1.6783*E* − 06	+	1.9016*E* − 06	+	1.6783*E* − 06	+	1.6783*E* − 06	+	1.6783*E* − 06	+	1.6783*E* − 06	+
**F8**	1.6783*E* − 06	+	3.5200*E* − 01	−	1.6783*E* − 06	+	1.6783*E* − 06	+	1.6783*E* − 06	+	3.6037*E* − 06	+
**F9**	*NA*	=	*NA*	=	1.6783*E* − 06	+	1.7626*E* − 06	+	1.6783*E* − 06	+	1.7516*E* − 06	+
**F10**	*NA*	=	*NA*	=	1.6783*E* − 06	+	1.7671*E* − 06	+	1.6783*E* − 06	+	1.6783*E* − 06	+
**F11**	*NA*	=	*NA*	=	2.0509*E* − 04	+	1.7671*E* − 06	+	1.6783*E* − 06	+	3.1250*E* − 02	+
**F12**	1.6783*E* − 06	+	1.7571*E* − 06	+	1.6783*E* − 06	+	2.6564*E* − 05	+	1.6783*E* − 06	+	1.7783*E* − 06	+
**F13**	1.6783*E* − 06	+	2.1912*E* − 05	+	1.6783*E* − 06	+	4.0773*E* − 01	−	1.6783*E* − 06	+	1.6783*E* − 06	+
**F14**	3.8754*E* − 06	+	3.1250*E* − 02	+	1.0385*E* − 06	+	*NA*	=	*NA*	=	2.8474*E* − 06	+
**F15**	1.6783*E* − 06	+	4.8468*E* − 06	+	1.5288*E* − 05	+	1.6783*E* − 06	+	1.6783*E* − 06	+	6.4874*E* − 06	+
**F16**	2.4136*E* − 06	+	9.9590*E* − 01	−	1.6783*E* − 06	+	1.6783*E* − 06	+	2.4136*E* − 06	+	1.9705*E* − 06	+
**F17**	2.9085*E* − 05	+	2.8861*E* − 02	+	2.1418*E* − 03	+	1.6783*E* − 06	+	2.1813*E* − 06	+	6.7653*E* − 04	+
**F18**	1.6783*E* − 06	+	2.6564*E* − 05	+	9.6309*E* − 01	−	1.3271*E* − 06	+	1.8777*E* − 02	+	2.3898*E* − 01	−
**F19**	2.4136*E* − 06	+	3.1106*E* − 01	−	2.2297*E* − 01	−	1.6783*E* − 06	+	1.6793*E* − 06	+	1.0309*E* − 01	−
**F20**	1.6783*E* − 06	+	5.3456*E* − 06	+	4.8468*E* − 06	+	5.3754*E* − 05	+	1.5105*E* − 03	+	3.8484*E* − 01	−
**F21**	1.9705*E* − 06	+	1.6764*E* − 02	+	1.6783*E* − 06	+	1.0308*E* − 01	−	1.6783*E* − 06	+	1.6783*E* − 06	+
**F22**	1.6783*E* − 06	+	5.4047*E* − 03	+	1.6783*E* − 06	+	3.7369*E* − 01	−	2.6697*E* − 06	+	1.6783*E* − 06	+
**F23**	4.8468*E* − 06	+	1.0041*E* + 00	−	1.6783*E* − 06	+	6.7704*E* − 01	−	2.1418*E* − 03	+	2.6663*E* − 04	+


Functions	SCA vs FROBLAO		LSHADE vs FROBLAO		CMA-ES vs FROBLAO		MRFO vs FROBLAO		AVOA vs FROBLAO	
	*p* − *cost*	*Hyp*	*p* − *cost*	*Hyp*	*p* − *cost*	*Hyp*	*p* − *cost*	*Hyp*	*p* − *cost*	*Hyp*
**F1**	1.6783*E* − 06	+	1.6783*E* − 06	+	1.6783*E* − 06	+	*NA*	=	2.0437*E* − 04	+
**F2**	1.6783*E* − 06	+	1.6783*E* − 06	+	1.6783*E* − 06	+	1.6783*E* − 06	+	1.6772*E* − 06	+
**F3**	1.6783*E* − 06	+	1.6783*E* − 06	+	1.6783*E* − 06	+	*NA*	=	1.6783*E* − 06	+
**F4**	1.6783*E* − 06	+	1.6783*E* − 06	+	1.6783*E* − 06	+	1.6783*E* − 06	+	1.7783*E* − 06	+
**F5**	1.6783*E* − 06	+	1.6783*E* − 06	+	1.6783*E* − 06	+	1.6783*E* − 06	+	1.6772*E* − 06	+
**F6**	1.6783*E* − 06	+	1.6783*E* − 06	+	1.6783*E* − 06	+	8.3142*E* − 07	+	1.6783*E* − 06	+
**F7**	1.6783*E* − 06	+	1.6783*E* − 06	+	1.6783*E* − 06	+	1.6783*E* − 06	+	1.7783*E* − 06	+
**F8**	3.6037*E* − 06	+	1.6783*E* − 06	+	1.6783*E* − 06	+	1.6783*E* − 06	+	1.7783*E* − 06	+
**F9**	2.6318*E* − 06	+	1.6783*E* − 06	+	1.6783*E* − 06	+	*NA*	=	*NA*	=
**F10**	1.6783*E* − 06	+	1.6726*E* − 06	+	1.6782*E* − 06	+	*NA*	=	*NA*	=
**F11**	1.6783*E* − 06	+	1.6783*E* − 06	+	1.6760*E* − 06	+	*NA*	=	*NA*	=
**F12**	1.6783*E* − 06	+	1.6783*E* − 06	+	1.6783*E* − 06	+	1.6783*E* − 06	+	5.3893*E* − 04	+
**F13**	1.6783*E* − 06	+	1.6783*E* − 06	+	1.6783*E* − 06	+	1.6446*E* − 06	+	7.3837*E* − 03	+
**F14**	1.7682*E* − 06	+	*NA*	=	1.6783*E* − 06	+	*NA*	=	7.8125*E* − 03	+
**F15**	1.6783*E* − 06	+	1.2097*E* − 02	+	1.6783*E* − 06	+	1.5360*E* − 01	−	2.6564*E* − 05	+
**F16**	1.1743*E* − 04	+	1.6783*E* − 06	+	1.6783*E* − 06	+	1.6783*E* − 06	+	1.6783*E* − 06	+
**F17**	1.9705*E* − 06	+	1.6783*E* − 06	+	1.6783*E* − 06	+	1.6783*E* − 06	+	1.7783*E* − 06	+
**F18**	3.0134*E* − 01	−	1.3271*E* − 06	+	1.3271*E* − 06	+	1.3271*E* − 06	+	1.6210*E* − 03	+
**F19**	2.1813*E* − 06	+	1.6783*E* − 06	+	1.6783*E* − 06	+	1.6783*E* − 06	+	1.7783*E* − 06	+
**F20**	1.6783*E* − 06	+	5.2385*E* − 02	−	9.9717*E* − 02	−	9.7917*E* − 01	−	9.7937*E* − 01	−
**F21**	1.6783*E* − 06	+	3.7369*E* − 01	−	3.7368*E* − 01	−	6.7704*E* − 01	−	1.7782*E* − 06	+
**F22**	1.6783*E* − 06	+	1.5007*E* − 02	+	1.6783*E* − 06	+	6.4721*E* − 01	−	1.7793*E* − 06	+
**F23**	1.6783*E* − 06	+	3.6599*E* − 04	+	3.1832*E* − 05	+	1.6660*E* − 01	−	1.7783*E* − 06	+

**Table 10:** Results on Wilcoxon rank-sum test for FROBLAO with other compared algorithms of CEC 2019 test functions.

Functions	AO vs FROBLAO		OBLAO vs FROBLAO		TSA vs FROBLAO		SSA vs FROBLAO		MVO vs FROBLAO		GWO vs FROBLAO	
	*p* − *cost*	*Hyp*	*p* − *cost*	*Hyp*	*p* − *cost*	*Hyp*	*p* − *cost*	*Hyp*	*p* − *cost*	*Hyp*	*p* − *cost*	*Hyp*
**CEC01**	1.6783*E* − 06	+	4.1210*E* − 02	+	1.9705*E* − 06	+	1.6783*E* − 06	+	1.6783*E* − 06	+	1.6783*E* − 06	+
**CEC02**	1.6783*E* − 06	+	7.6771*E* − 05	+	1.6783*E* − 06	+	2.8122*E* − 03	+	1.6783*E* − 06	+	1.6783*E* − 06	+
**CEC03**	1.6783*E* − 06	+	1.6783*E* − 06	+	5.4603*E* − 06	+	1.6726*E* − 06	+	1.6726*E* − 06	+	1.5004*E* − 02	+
**CEC04**	1.6783*E* − 06	+	1.7299*E* − 01	−	1.6783*E* − 06	+	6.4721*E* − 01	−	2.4728*E* − 01	−	2.7387*E* − 02	+
**CEC05**	1.9705*E* − 06	+	2.0041*E* − 01	−	1.6783*E* − 06	+	8.0107*E* − 01	−	5.0718*E* − 03	−	1.7390*E* − 03	+
**CEC06**	1.6783*E* − 06	+	2.5802*E* − 06	+	1.6783*E* − 06	+	1.0348*E* − 02	+	2.4136*E* − 06	+	2.4136*E* − 06	+
**CEC07**	3.9795*E* − 06	+	1.6210*E* − 03	+	1.6783*E* − 06	+	5.9202*E* − 05	+	5.8934*E* − 06	+	5.3456*E* − 06	+
**CEC08**	2.4136*E* − 06	+	1.2642*E* − 05	+	1.6783*E* − 06	+	2.4136*E* − 06	+	1.9705*E* − 06	+	2.9085*E* − 05	+
**CEC09**	1.6783*E* − 06	+	7.8780*E* − 06	+	1.6783*E* − 06	+	2.1813*E* − 06	+	3.6037*E* − 06	+	1.6783*E* − 06	+
**CEC10**	1.6783*E* − 06	+	1.2634*E* − 02	+	1.6783*E* − 06	+	1.1535*E* − 05	+	3.9795*E* − 06	+	1.6783*E* − 06	+


Functions	SCA vs FROBLAO		LSHADE vs FROBLAO		CMA-ES vs FROBLAO		MRFO vs FROBLAO		AVOA vs FROBLAO	
	*p* − *cost*	*Hyp*	*p* − *cost*	*Hyp*	*p* − *cost*	*Hyp*	*p* − *cost*	*Hyp*	*p* − *cost*	*Hyp*
**CEC01**	1.6783*E* − 06	+	1.6783*E* − 06	+	1.6783*E* − 06	+	1.6783*E* − 06	+	3.4825*E* − 05	+
**CEC02**	1.6783*E* − 06	+	1.4299*E* − 06	+	1.6783*E* − 06	+	1.4299*E* − 06	+	1.7505*E* − 06	+
**CEC03**	1.6783*E* − 06	+	1.6726*E* − 06	+	1.6783*E* − 06	+	8.3083*E* − 07	+	9.3629*E* − 07	+
**CEC04**	1.6783*E* − 06	+	1.6783*E* − 06	+	7.1545*E* − 06	+	9.9897*E* − 03	+	9.9897*E* − 03	+
**CEC05**	1.6783*E* − 06	+	1.9705*E* − 06	+	1.6783*E* − 06	+	7.3837*E* − 03	+	2.2941*E* − 03	+
**CEC06**	1.6783*E* − 06	+	2.1293*E* − 06	+	1.6783*E* − 06	+	1.6783*E* − 06	+	1.6783*E* − 06	+
**CEC07**	1.6783*E* − 06	+	2.5855*E* − 05	+	8.3144*E* − 05	+	5.4246*E* − 05	+	2.1813*E* − 06	+
**CEC08**	1.6783*E* − 06	+	6.4947*E* − 06	+	1.6774*E* − 04	+	1.2770*E* − 04	+	1.9705*E* − 06	+
**CEC09**	1.6783*E* − 06	+	5.8934*E* − 06	+	5.3754*E* − 05	+	7.6060*E* − 02	+	1.6783*E* − 06	+
**CEC10**	1.6783*E* − 06	+	2.6697*E* − 06	+	1.6783*E* − 06	+	2.4608*E* − 04	+	7.1545*E* − 06	+

**Table 11:** Results on Wilcoxon rank-sum test for FROBLAO with other compared algorithms of CEC 2020 test functions.

Functions	AO vs FROBLAO		OBLAO vs FROBLAO		TSA vs FROBLAO		SSA vs FROBLAO		MVO vs FROBLAO		GWO vs FROBLAO	
	*p* − *cost*	*Hyp*	*p* − *cost*	*Hyp*	*p* − *cost*	*Hyp*	*p* − *cost*	*Hyp*	*p* − *cost*	*Hyp*	*p* − *cost*	*Hyp*
**H1**	1.6783*E* − 06	+	4.6220*E* − 04	+	1.6783*E* − 06	+	2.1813*E* − 06	+	1.6783*E* − 06	+	4.9685*E* − 05	+
**H2**	2.4136*E* − 06	+	1.5082*E* − 04	+	1.6783*E* − 06	+	7.8780*E* − 06	+	1.6780*E* − 05	+	6.2739*E* − 04	+
**H3**	1.6783*E* − 06	+	1.1535*E* − 05	+	1.6783*E* − 06	+	1.5082*E* − 04	+	1.6783*E* − 06	+	2.6287*E* − 03	+
**H4**	1.6783*E* − 06	+	1.6783*E* − 06	+	1.6783*E* − 06	+	1.6783*E* − 06	+	1.6783*E* − 06	+	1.6783*E* − 06	+
**H5**	1.6783*E* − 06	+	1.7079*E* − 06	+	1.6783*E* − 06	+	1.6783*E* − 06	+	1.6783*E* − 06	+	1.6783*E* − 06	+
**H6**	1.6783*E* − 06	+	1.6783*E* − 06	+	1.6783*E* − 06	+	1.6783*E* − 06	+	1.9705*E* − 06	+	1.6783*E* − 06	+
**H7**	1.6783*E* − 06	+	1.6783*E* − 06	+	1.6783*E* − 06	+	1.6783*E* − 06	+	1.6783*E* − 06	+	1.6783*E* − 06	+
**H8**	3.1832*E* − 05	+	4.9685*E* − 05	+	2.9517*E* − 06	+	2.9085*E* − 05	+	7.8780*E* − 06	+	1.9705*E* − 06	+
**H9**	2.2132*E* − 05	+	2.4608*E* − 04	+	1.6783*E* − 06	+	1.2676*E* − 05	+	2.9085*E* − 05	+	7.1545*E* − 06	+
**H10**	6.9500*E* − 02	+	1.6783*E* − 06	+	7.6771*E* − 05	+	1.6783*E* − 06	+	8.3117*E* − 02	+	1.6783*E* − 06	+


Functions	SCA vs FROBLAO		LSHADE vs FROBLAO		CMA-ES vs FROBLAO		MRFO vs FROBLAO		AVOA vs FROBLAO	
	*p* − *value*	*Hyp*	*p* − *value*	*Hyp*	*p* − *value*	*Hyp*	*p* − *value*	*Hyp*	*p* − *value*	*Hyp*
**H1**	1.7783*E* − 06	+	1.6783*E* − 06	+	2.9517*E* − 06	+	1.6783*E* − 06	+	1.6783*E* − 06	+
**H2**	1.6783*E* − 06	+	4.4401*E* − 03	+	1.6783*E* − 06	+	6.4947*E* − 06	+	1.6783*E* − 06	+
**H3**	1.6783*E* − 06	+	1.6783*E* − 06	+	2.3842*E* − 06	+	1.3924*E* − 05	+	3.2621*E* − 06	+
**H4**	1.6783*E* − 06	+	2.9250*E* − 06	+	1.6783*E* − 06	+	1.6783*E* − 06	+	1.6783*E* − 06	+
**H5**	1.6783*E* − 06	+	1.3348*E* − 04	+	1.6783*E* − 06	+	1.6783*E* − 06	+	1.6783*E* − 06	+
**H6**	1.6783*E* − 06	+	1.3881*E* − 04	+	1.9705*E* − 06	+	7.0429*E* − 05	+	2.1813*E* − 06	+
**H7**	1.6783*E* − 06	+	2.6318*E* − 06	+	1.6783*E* − 06	+	1.6783*E* − 06	+	1.6783*E* − 06	+
**H8**	1.9705*E* − 06	+	4.9958*E* − 01	−	1.5625*E* − 02	−	6.3412*E* − 02	+	4.2997*E* − 07	+
**H9**	1.6783*E* − 06	+	2.1587*E* − 05	+	7.8780*E* − 06	+	4.4506*E* − 03	+	3.1250*E* − 02	−
**H10**	2.1813*E* − 06	+	1.6783*E* − 06	+	1.6783*E* − 06	+	1.6783*E* − 06	+	1.6783*E* − 06	+

• **t-test:** Two-tailed *t*-tests [12] has been used to compare different statistical outcomes at a consequence of 0.05. The *t* values are determined with the help of Mean and *Std* values. A negative value illustrates that the statistical results of the FROBLAO optimization mistakes are significantly less, and vice versa. The corresponding t−cost is highlighted if the difference is a statistically significant error. The symbols +/=/− represent that FROBLAO wins functions, ties functions, and loss functions. However, Table 12 shows the t-test outcomes on CEC 2005 test functions, Table 13 shows the t-test results on CEC 2019 test functions, and Table 14 shows the t-test results on CEC 2020 test functions. The statistical outcomes of the optimization mistakes demonstrate that FROBLAO has much superior total achievement when compared with the other algorithms.

**Table 12:** Results on *t*-test for FROBLAO with other compared algorithms of CEC 2005 test functions.

Functions	AO vs FROBLAO		OBLAO vs FROBLAO		TSA vs FROBLAO		SSA vs FROBLAO		MVO vs FROBLAO		GWO vs FROBLAO	
	*t* − *cost*	+/ = /−	*t* − *cost*	+/ = /−	*t* − *cost*	+/ = /−	*t* − *cost*	+/ = /−	*t* − *cost*	+/ = /−	*t* − *cost*	+/ = /−
**F1**	−1.000*E* + 00	=	0	=	−2.108*E* + 00	+	−4.580*E* + 00	+	−1.065*E* + 01	+	−2.834*E* + 00	+
**F2**	−1.001*E* + 00	=	0	=	−5.377*E* + 00	+	−1.981*E* + 00	+	−1.490*E* + 01	+	−8.452*E* + 00	+
**F3**	−1.001*E* + 00	=	0	=	−2.340*E* + 00	+	−1.907*E* + 00	+	−8.989*E* + 00	+	−1.881*E* + 00	+
**F4**	−1.001*E* + 00	=	0	=	−4.684*E* + 00	+	−2.064*E* + 00	+	−1.936*E* + 01	+	−3.865*E* + 00	+
**F5**	−4.100*E* + 00	+	−6.257*E* + 00	+	−1.616*E* + 02	+	−1.900*E* + 00	+	−2.708*E* + 00	+	−1.796*E* + 02	+
**F6**	−3.814*E* + 00	+	−5.336*E* + 00	+	−2.633*E* + 01	+	2.994*E* + 00	−	−1.217*E* + 01	+	−1.118*E* + 01	+
**F7**	−4.319*E* + 00	+	−8.799*E* + 00	+	−1.035*E* + 01	+	−9.442*E* + 00	+	−7.119*E* + 00	+	−1.051*E* + 01	+
**F8**	−3.807*E* + 00	+	8.229*E* − 01	=	1.403*E* + 01	−	−2.655*E* + 01	+	−2.129*E* + 01	+	9.217*E* + 00	−
**F9**	0	=	0	=	−2.146*E* + 01	+	−1.161*E* + 01	+	−1.356*E* + 01	+	−5.079*E* + 00	+
**F10**	0	=	0	=	−5.679*E* + 00	+	−4.114*E* + 00	+	−4.991*E* + 00	+	−2.901*E* + 01	+
**F11**	0	=	0	=	−6.020*E* + 00	+	−1.016*E* + 01	+	−1.526*E* + 01	+	−2.361*E* + 00	+
**F12**	−2.267*E* + 00	+	−6.770*E* + 00	+	−1.208*E* + 01	+	−3.872*E* + 00	+	−1.838*E* + 00	+	−1.238*E* + 01	+
**F13**	−2.325*E* + 00	+	−5.665*E* + 00	+	−2.727*E* + 01	+	−1.996*E* + 00	+	−6.764*E* + 00	+	−1.532*E* + 01	+
**F14**	−2.975*E* + 00	+	−2.693*E* + 00	+	−1.013*E* + 01	+	−2.250*E* + 00	+	2.903*E* + 00	−	−5.487*E* + 00	+
**F15**	−9.310*E* + 00	+	−6.719*E* + 00	+	−2.538*E* + 00	+	−2.776*E* + 00	+	−2.940*E* + 00	+	−3.306*E* + 00	+
**F16**	−5.571*E* + 00	+	6.469*E* − 01	=	4.221*E* + 00	−	4.389*E* + 00	−	4.260*E* + 00	−	4.381*E* + 00	−
**F17**	−3.081*E* + 00	+	2.373*E* + 00	−	−3.607*E* + 00	+	4.985*E* + 00	−	4.675*E* + 00	−	−8.884*E* − 02	=
**F18**	−5.571*E* + 00	+	−4.508*E* + 00	+	−2.044*E* + 00	+	3.126*E* + 00	−	−9.999*E* − 01	=	2.554*E* + 00	−
**F19**	−7.584*E* + 00	+	−8.230*E* − 01	=	−6.820*E* − 01	=	4.971*E* + 00	−	4.940*E* + 00	−	−3.235*E* + 00	+
**F20**	−1.168*E* + 01	+	−7.920*E* + 00	+	−5.061*E* + 00	+	−8.578*E* + 00	+	−6.382*E* + 00	+	−3.326*E* + 00	+
**F21**	−2.608*E* + 00	+	−2.051*E* + 00	+	−8.916*E* + 00	+	−4.566*E* + 00	+	−5.534*E* + 00	+	−3.437*E* + 00	+
**F22**	−3.860*E* + 00	+	−3.090*E* + 00	+	−6.126*E* + 00	+	−2.931*E* + 00	+	−4.020*E* + 00	+	−1.123*E* + 01	+
**F23**	−2.732*E* + 00	+	1.002*E* + 00	=	−5.808*E* + 00	+	−3.215*E* + 00	+	−3.708*E* + 00	+	−3.971*E* + 00	+


Functions	SCA vs FROBLAO		LSHADE vs FROBLAO		CMA-ES vs FROBLAO		MRFO vs FROBLAO		AVOA vs FROBLAO	
	*t* − *cost*	+/ = /−	*t* − *cost*	+/ = /−	*t* − *cost*	+/ = /−	*t* − *cost*	+/ = /−	*t* − *cost*	+/ = /−
**F1**	−1.402*E* + 00	+	−3.956*E* + 00	+	−1.349*E* + 01	+	0	=	0	=
**F2**	−2.421*E* + 00	+	−4.999*E* + 00	+	−1.839*E* + 01	+	0	=	−1.0003*E* + 00	=
**F3**	−1.358*E* + 00	+	−8.727*E* + 00	+	−1.087*E* + 01	+	0	=	0	=
**F4**	−2.555*E* + 00	+	−2.608*E* + 01	+	−2.051*E* + 01	+	0	=	−1.0016*E* + 00	=
**F5**	−9.725*E* + 01	+	−7.408*E* + 00	+	−5.497*E* + 00	+	−2.6490*E* + 02	+	−6.8573*E* + 00	+
**F6**	−1.354*E* + 01	+	−6.243*E* + 00	+	−1.479*E* + 01	+	3.2213*E* + 00	−	−9.3385*E* + 00	+
**F7**	−7.784*E* + 00	+	−1.166*E* + 01	+	−2.043*E* + 01	+	−5.8423*E* + 00	+	−6.1218*E* + 00	+
**F8**	−6.238*E* + 01	+	2.211*E* + 02	−	9.481*E* + 00	−	2.9169*E* + 01	−	4.7934*E* + 01	−
**F9**	−1.184*E* + 00	+	−1.166*E* + 01	+	−1.013*E* + 01	+	0	=	0	=
**F10**	−1.003*E* + 00	=	−1.909*E* + 01	+	−2.304*E* + 01	+	0	=	0	=
**F11**	−3.940*E* + 00	+	−6.692*E* + 00	+	−1.636*E* + 01	+	0	=	0	=
**F12**	−1.553*E* + 01	+	−6.010*E* + 00	+	−1.212*E* + 01	+	2.4974*E* + 00	−	−3.0426*E* + 00	+
**F13**	−1.744*E* + 01	+	−4.424*E* + 00	+	−1.416*E* + 01	+	−1.1800*E* + 01	+	−3.2659*E* + 00	+
**F14**	−2.580*E* + 00	+	4.731*E* + 00	−	−7.106*E* + 00	+	4.7309*E* + 00	−	−2.9064*E* + 00	+
**F15**	−1.151*E* + 01	+	−2.040*E* + 00	+	−8.345*E* + 00	+	−2.1723*E* + 00	+	−3.1245*E* + 00	+
**F16**	−3.271*E* + 00	+	4.389*E* + 00	−	4.389*E* + 00	−	4.3895*E* + 00	−	4.3895*E* + 00	−
**F17**	−5.395*E* + 00	+	4.985*E* + 00	−	4.985*E* + 00	−	4.9855*E* + 00	−	4.9855*E* + 00	−
**F18**	1.706*E* + 00	−	3.126*E* + 00	−	3.126*E* + 00	−	3.1264*E* + 00	−	3.0742*E* + 00	−
**F19**	−1.337*E* + 01	+	−7.388*E* + 04	+	4.971*E* + 00	−	4.9708*E* + 00	−	4.9708*E* + 00	−
**F20**	−6.260*E* + 00	+	−2.127*E* + 00	+	−4.497*E* + 00	+	−3.2965*E* + 00	+	−3.2980*E* + 00	+
**F21**	−1.785*E* + 01	+	−2.906*E* + 00	+	−2.971*E* + 00	+	−3.2473*E* + 00	+	3.3111*E* + 00	−
**F22**	−1.942*E* + 01	+	−2.075*E* + 00	+	1.777*E* + 01	−	−3.8076*E* + 00	+	1.7770*E* + 01	−
**F23**	−2.316*E* + 01	+	−1.428*E* + 00	+	−9.980*E* − 01	=	−2.6912*E* + 00	+	2.9390*E* + 00	−

**Table 13:** Results on *t*-test for FROBLAO with other compared algorithms of CEC 2019 test functions.

Functions	AO vs FROBLAO		OBLAO vs FROBLAO		TSA vs FROBLAO		SSA vs FROBLAO		MVO vs FROBLAO		GWO vs FROBLAO	
	*t* − *cost*	+/ = /−	*t* − *cost*	+/ = /−	*t* − *cost*	+/ = /−	*t* − *cost*	+/ = /−	*t* − *cost*	+/ = /−	*t* − *cost*	+/ = /−
**CEC01**	−9.1963*E* + 00	+	−9.6403*E* + 00	+	−2.6913*E* + 00	+	−4.6593*E* + 00	+	−7.5488*E* + 00	+	−2.7393*E* + 00	+
**CEC02**	−1.2778*E* + 01	+	−1.2787*E* + 01	+	−9.3760*E* + 00	+	−1.7762*E* + 00	+	−1.1091*E* + 01	+	−1.6805*E* + 01	+
**CEC03**	−7.0577*E* + 00	+	−9.4426*E* + 00	+	−2.4577*E* + 00	+	−1.0312*E* + 00	+	−6.6376*E* + 00	+	−1.7183*E* + 00	+
**CEC04**	−6.1907*E* + 00	+	−1.2611*E* + 00	+	−8.2229*E* + 00	+	−5.7366*E* − 01	=	1.2853*E* + 00	−	−1.3379*E* + 00	+
**CEC05**	−9.2935*E* + 00	+	−1.5279*E* + 00	+	−1.3751*E* + 01	+	−7.2125*E* − 01	=	−3.1095*E* + 00	+	−4.1248*E* + 00	+
**CEC06**	−1.8494*E* + 01	+	−1.0910*E* + 01	+	−2.6692*E* + 01	+	−1.8824*E* + 00	+	−9.9515*E* + 00	+	−2.6159*E* + 01	+
**CEC07**	−8.7188*E* + 00	+	−3.2101*E* + 00	+	−1.3526*E* + 01	+	−5.8691*E* + 00	+	−5.9688*E* + 00	+	−7.3466*E* + 00	+
**CEC08**	−1.0844*E* + 01	+	−6.8359*E* + 00	+	−1.6670*E* + 01	+	−9.4979*E* + 00	+	−9.0220*E* + 00	+	−6.3188*E* + 00	+
**CEC09**	−1.8056*E* + 01	+	−7.8734*E* + 00	+	−4.7217*E* + 00	+	−1.0134*E* + 01	+	−1.0049*E* + 01	+	−1.4754*E* + 01	+
**CEC10**	−8.6813*E* + 00	+	−3.1040*E* + 00	+	−1.1046*E* + 01	+	−1.0721*E* + 01	+	−1.0767*E* + 01	+	−1.1019*E* + 01	+


Functions	SCA vs FROBLAO		LSHADE vs FROBLAO		CMA-ES vs FROBLAO		MRFO vs FROBLAO		AVOA vs FROBLAO	
	*t* − *cost*	+/ = /−	*t* − *cost*	+/ = /−	*t* − *cost*	+/ = /−	*t* − *cost*	+/ = /−	*t* − *cost*	+/ = /−
**CEC01**	−4.0141*E* + 00	+	−2.0904*E* + 00	+	−7.1166*E* + 00	+	−3.2162*E* + 00	+	−1.0964*E* + 01	+
**CEC02**	−9.2177*E* + 00	+	−2.2622*E* + 00	+	−1.0598*E* + 01	+	−2.9000*E* + 01	+	−2.8330*E* + 00	+
**CEC03**	−5.2516*E* + 00	+	0	=	−2.0604*E* + 00	+	0	=	−1.0002*E* + 00	=
**CEC04**	−9.5271*E* + 00	+	9.0309*E* + 00	−	5.5973*E* + 00	−	2.0776*E* + 00	−	−7.4697*E* + 00	+
**CEC05**	−3.9844*E* + 01	+	1.0115*E* + 01	−	1.0280*E* + 01	−	2.6750*E* + 00	−	−3.4974*E* + 00	+
**CEC06**	−2.4801*E* + 01	+	−7.7271*E* + 00	+	−2.3426*E* + 01	+	−2.2247*E* + 01	+	−1.9168*E* + 00	+
**CEC07**	−2.1418*E* + 01	+	−5.8694*E* + 00	+	−5.0410*E* + 00	+	−6.1675*E* + 00	+	−7.6934*E* + 00	+
**CEC08**	−1.6843*E* + 01	+	−6.9555*E* + 00	+	−5.0856*E* + 00	+	−5.9736*E* + 00	+	−1.3848*E* + 01	+
**CEC09**	−6.5860*E* + 00	+	−5.5750*E* + 00	+	−1.5600*E* + 01	+	−2.9052*E* + 00	+	−1.1004*E* + 01	+
**CEC10**	−1.1048*E* + 01	+	−1.0762*E* + 01	+	−1.0996*E* + 01	+	−5.7163*E* + 00	+	−1.0726*E* + 01	+

**Table 14:** Results on *t*-test for FROBLAO with other compared algorithms of CEC 2020 test functions.

Functions	AO vs FROBLAO		OBLAO vs FROBLAO		TSA vs FROBLAO		SSA vs FROBLAO		MVO vs FROBLAO		GWO vs FROBLAO	
	*t* − *cost*	+/ = /−	*t* − *cost*	+/ = /−	*t* − *cost*	+/ = /−	*t* − *cost*	+/ = /−	*t* − *cost*	+/ = /−	*t* − *cost*	+/ = /−
**H1**	−2.2912*E* + 00	+	−4.0711*E* + 00	+	−6.1688*E* + 00	+	5.7111*E* + 00	−	5.4235*E* + 00	−	−1.8943*E* + 00	+
**H2**	−8.6425*E* + 00	+	−5.1919*E* + 00	+	−1.5611*E* + 01	+	−6.9648*E* + 00	+	−5.5446*E* + 00	+	−3.9755*E* + 00	+
**H3**	−1.1080*E* + 01	+	−6.6810*E* + 00	+	−1.4511*E* + 01	+	−4.3347*E* + 00	+	−2.4907*E* + 00	+	−3.9553*E* + 00	+
**H4**	−3.4453*E* + 00	+	−2.7717*E* + 00	+	−2.7868*E* + 00	+	−3.9362*E* + 00	+	−3.3731*E* + 00	+	−5.7090*E* + 00	+
**H5**	−2.2592*E* + 01	+	−8.3911*E* + 00	+	−6.3279*E* + 00	+	−3.4348*E* + 00	+	−5.5701*E* + 00	+	−2.2272*E* + 00	+
**H6**	−8.3700*E* + 00	+	−3.9279*E* + 00	+	−1.6070*E* + 01	+	−9.9252*E* + 00	+	−7.4720*E* + 00	+	−6.8687*E* + 00	+
**H7**	−6.4387*E* + 00	+	−6.4067*E* + 00	+	−2.8574*E* + 00	+	−4.3024*E* + 00	+	−5.5972*E* + 00	+	−6.9381*E* + 00	+
**H8**	−4.1360*E* + 00	+	−1.1112*E* + 00	+	−5.3102*E* + 00	+	−1.0761*E* + 00	+	−1.1279*E* + 00	+	−6.9509*E* + 00	+
**H9**	−6.3795*E* + 00	+	−5.3496*E* + 00	+	−1.5694*E* + 01	+	−6.9008*E* + 00	+	−6.2250*E* + 00	+	−1.0522*E* + 01	+
**H10**	−8.5002*E* − 01	+	1.3268*E* + 01	−	−4.8284*E* + 00	+	−1.9492*E* + 00	+	1.8424*E* + 00	−	−1.9300*E* + 00	+


Functions	SCA vs FROBLAO		LSHADE vs FROBLAO		CMA-ES vs FROBLAO		MRFO vs FROBLAO		AVOA vs FROBLAO	
	*t* − *cost*	+/ = /−	*t* − *cost*	+/ = /−	*t* − *cost*	+/ = /−	*t* − *cost*	+/ = /−	*t* − *cost*	+/ = /−
**H1**	−1.7200*E* + 01	+	1.1266*E* + 01	−	−4.4030*E* + 00	+	1.0521*E* + 01	−	1.0876*E* + 01	−
**H2**	−2.7658*E* + 01	+	−3.6651*E* + 00	+	−2.2161*E* + 01	+	−7.7591*E* + 00	+	−9.4395*E* + 00	+
**H3**	−2.3658*E* + 01	+	3.1439*E* + 00	−	−8.6408*E* + 00	+	−6.3381*E* + 00	+	−8.8609*E* + 00	+
**H4**	−7.7271*E* + 00	+	−9.9863*E* + 00	+	−4.8987*E* + 00	+	−5.6806*E* + 00	+	−3.6755*E* + 00	+
**H5**	−4.6582*E* + 00	+	−6.0743*E* + 00	+	−3.9980*E* + 00	+	−6.1530*E* + 00	+	−4.4354*E* + 00	+
**H6**	−1.0858*E* + 01	+	−2.7441*E* + 00	+	−6.8386*E* + 00	+	−4.6564*E* + 00	+	−6.3569*E* + 00	+
**H7**	−9.3950*E* + 00	+	−4.1850*E* + 00	+	−4.8858*E* + 00	+	−6.4080*E* + 00	+	−4.7118*E* + 00	+
**H8**	−1.0456*E* + 01	+	2.8455*E* + 00	−	2.8455*E* + 00	−	5.6207*E* − 01	=	−1.5235*E* + 02	+
**H9**	−1.3825*E* + 01	+	−6.4329*E* + 00	+	−1.0670*E* + 01	+	−3.2098*E* + 00	+	2.6803*E* + 00	−
**H10**	−8.8717*E* + 00	+	−6.5339*E* − 01	=	−8.0680*E* − 01	=	1.0457*E* + 00	=	5.3863*E* + 01	−

• **Friedman test:** The Friedman test [89] is used to further highlight how each of the 11 competing algorithms performed overall. The algorithms can be ranked using the Friedman test according to how well they handle each problem independently. The performance rank of each algorithm for CEC2005, CEC2019, and CEC2020 benchmark functions is shown in Table 15, Table 16, Table 17, respectively. From Table 15, the mean rank of FROBLAO is 1.52173913, which indicates FROBLAO has the best overall performance in dealing with CEC2005 test functions. According to Table 16, FROBLAO has the finest comprehensive performance in dealing with CEC2019 test functions, with a mean rank of 1.6. Table 17 shows that FROBLAO has the best comprehensive performance in dealing with CEC2020 test functions, with a mean rank of 2.4. The Friedman test results once again demonstrate FROBLAO's superiority over the other optimizers analyzed.

**Table 15:** Performance rank of each algorithm for CEC 2005 test functions.

Functions	*AO*	*OBLAO*	*TSA*	*SSA*	*MVO*	*GWO*	*SCA*	*LSHADE*	*CMA* − *ES*	*MRFO*	*AVOA*	*FROBLAO*
**F1**	5	2	7	9	11	6	8	12	10	3	4	1
**F2**	5	2	7	10	11	6	8	12	9	3	4	1
**F3**	5	2	8	6	10	7	9	12	11	3	4	1
**F4**	5	2	11	7	10	6	8	12	9	3	4	1
**F5**	4	2	8	11	12	7	5	10	9	6	3	1
**F6**	7	5	12	2	8	11	10	9	6	1	4	3
**F7**	3	2	9	10	8	6	7	12	11	4	5	1
**F8**	9	8	6	11	10	5	12	3	1	4	2	7
**F9**	5	2	11	10	9	7	6	8	12	4	3	1
**F10**	4	3	11	10	9	6	8	12	7	5	2	1
**F11**	3	2	8	11	12	7	9	10	6	5	4	1
**F12**	6	4	12	10	7	8	9	11	5	2	3	1
**F13**	5	3	12	6	7	9	8	11	4	10	2	1
**F14**	9	6	12	5	3	10	8	2	11	4	7	1
**F15**	6	2	12	9	8	7	11	4	10	5	3	1
**F16**	12	10	8	6	9	7	11	2	3	5	4	1
**F17**	11	8	9	6	7	10	12	2	4	3	5	1
**F18**	10	9	12	5	11	7	8	3	4	2	6	1
**F19**	11	7	8	4	6	9	10	12	2	1	3	5
**F20**	11	9	6	10	8	7	12	2	5	3	4	1
**F21**	4	3	11	9	10	8	12	6	7	5	2	1
**F22**	6	4	11	8	10	5	12	7	2	9	3	1
**F23**	5	2	11	9	10	4	12	7	6	8	3	1
Sum of ranks	151	99	222	184	206	165	215	181	154	98	84	35
Mean of ranks	6.565217391	4.304347826	9.652173913	8	8.956521739	7.173913043	9.347826087	7.869565217	6.695652174	4.260869565	3.652173913	1.52173913
Overall ranks	5	4	12	9	10	7	11	8	6	3	2	1

**Table 16:** Performance rank of each algorithm for CEC 2019 test functions.

Functions	*AO*	*OBLAO*	*TSA*	*SSA*	*MVO*	*GWO*	*SCA*	*LSHADE*	*CMA* − *ES*	*MRFO*	*AVOA*	*FROBLAO*
**CEC01**	5	3	9	11	10	7	12	6	8	2	4	1
**CEC02**	7	6	10	8	11	4	9	2	12	5	3	1
**CEC03**	9	7	12	4	6	8	10	2	11	3	5	1
**CEC04**	10	7	12	6	4	9	11	1	2	3	8	5
**CEC05**	10	6	12	5	7	8	11	2	1	4	9	3
**CEC06**	9	4	12	2	5	11	10	6	8	7	3	1
**CEC07**	9	2	12	6	7	10	11	4	5	3	8	1
**CEC08**	9	2	12	6	8	5	11	3	7	4	10	1
**CEC09**	10	6	12	7	5	9	11	4	3	2	8	1
**CEC10**	4	2	9	5	8	12	10	7	11	3	6	1
Sum of ranks	82	45	112	60	71	83	106	37	68	36	64	16
Mean of ranks	8.2	4.5	11.2	6	7.1	8.3	10.6	3.7	6.8	3.6	6.4	1.6
Overall ranks	9	4	12	5	8	10	11	3	7	2	6	1

**Table 17:** Performance rank of each algorithm for CEC 2020 test functions.

Functions	*AO*	*OBLAO*	*TSA*	*SSA*	*MVO*	*GWO*	*SCA*	*LSHADE*	*CMA* − *ES*	*MRFO*	*AVOA*	*FROBLAO*
**H1**	10	7	12	4	5	9	11	1	8	3	2	6
**H2**	6	3	10	9	5	4	11	2	12	7	8	1
**H3**	9	7	12	5	3	4	11	1	6	8	10	2
**H4**	10	4	12	11	6	7	9	2	8	3	5	1
**H5**	6	5	11	7	4	9	10	2	12	3	8	1
**H6**	10	4	12	11	8	7	9	2	5	6	3	1
**H7**	10	4	12	8	6	7	9	2	11	3	5	1
**H8**	6	5	12	9	8	7	11	3	2	1	10	4
**H9**	7	4	12	6	5	8	11	10	9	3	1	2
**H10**	8	2	12	10	4	9	11	7	6	3	1	5
Sum of ranks	82	45	117	80	54	71	103	32	79	40	53	24
Mean of ranks	8.2	4.5	11.7	8	5.4	7.1	10.3	3.2	7.9	4	5.3	2.4
Overall ranks	10	4	12	9	6	7	11	2	8	3	5	1

### 3.7 Convergence curve analysis

**Figure 8:**
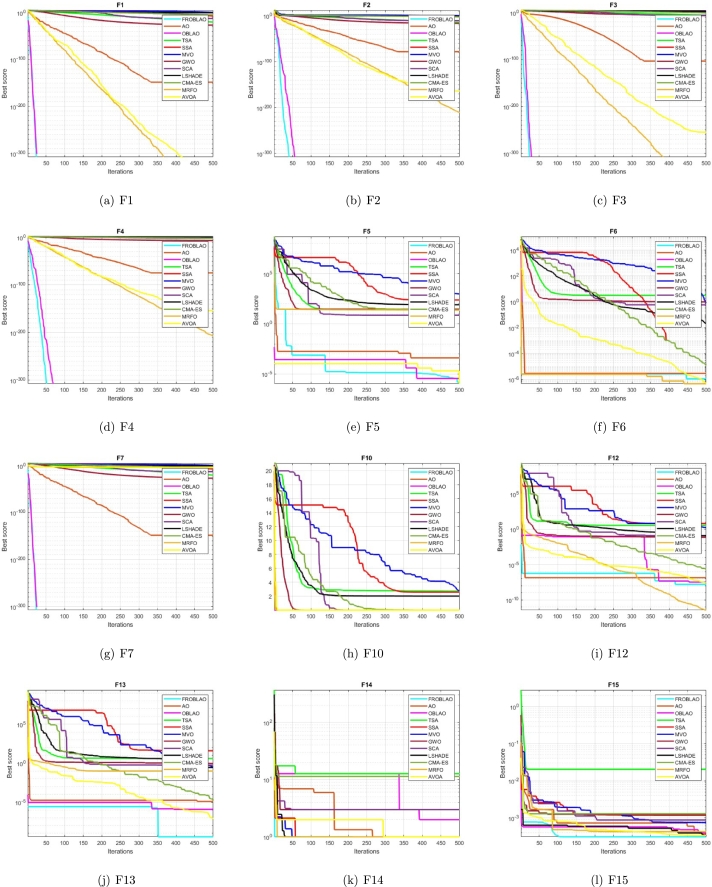

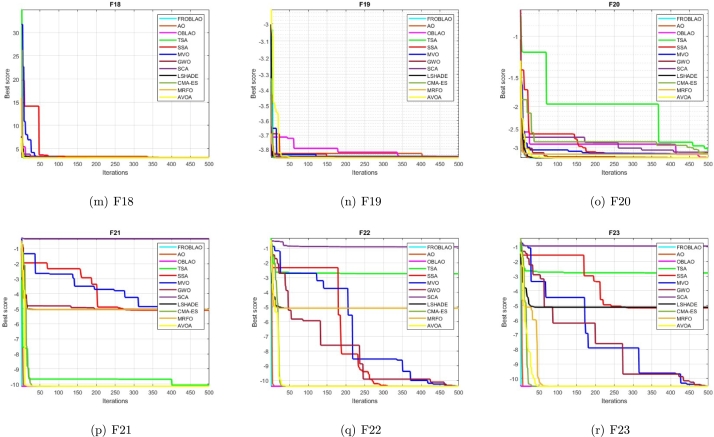
Convergence curves of FROBLAO algorithm in comparison to other algorithms on CEC 2005 test functions.

**Figure 9:**
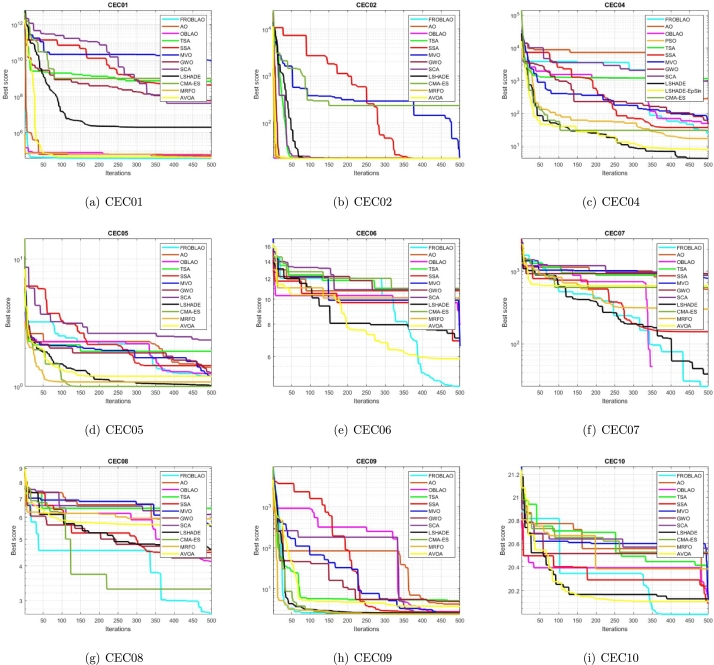
Convergence curves of FROBLAO algorithm in comparison to other algorithms on CEC 2019 test functions.

**Figure 10:**
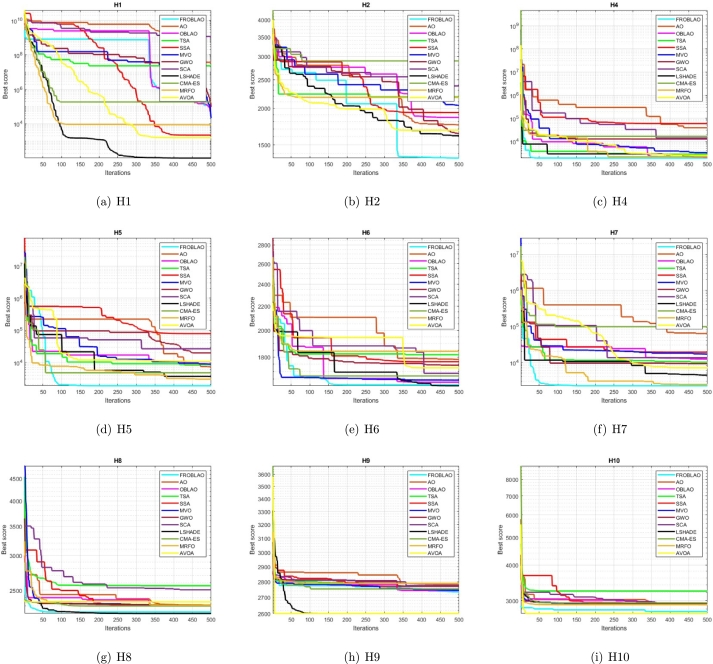
Convergence curves of FROBLAO algorithm in comparison to other algorithms on CEC 2020 test functions.

The convergence curve demonstrates the relationship between the fitness function solution and the maximum number of iterations. In the early stages of the optimization process, the population investigates the search region and deviates significantly. The major motive behind the convergence investigation is to understand the behavior and graphical explanation of the FROBLAO. Figs. 8(a)-8(r) show the convergence curves of the all-compared algorithms for the CEC 2005 test functions. From, Fig. 8 it is noticed that the proposed method FROBLAO converges faster among all objective functions except for *F*6, *F*12, and *F*19. However, the FROBLAO algorithm has a larger impact on the convergence of the other algorithms, especially compared with the AO, OBLAO, TSA, SSA, MVO, GWO, SCA, LSHADE, CMA-ES, MRFO, and AVOA. Figs. 9(a)-9(i) show the convergence curve of all the compared algorithms for the CEC 2019 test functions. Fig. 9 shows that the FROBLAO algorithm converges quicker than all fitness functions left of CEC04, and CEC05. Fig. 9 demonstrates that the proposed FROBLAO method outperforms existing algorithms in terms of convergence. Figs. 10(a)-10(i) show the convergence curve of all the compared algorithms for the CEC 2020 test functions. Fig. 10 shows that the FROBLAO algorithm converges quicker than all fitness functions left of *H*1, *H*9, and *H*10. Fig. 10 demonstrates that the proposed FROBLAO method outperforms existing algorithms in terms of convergence. Thus, it is demonstrated that enhancements to the suggested FROBLAO method can result in improved quality of searches and quicker convergence of the graph.

## 4 FROBLAO for solving real-life engineering problems

This section evaluates the proposed algorithm's performance in six real-life engineering problems using constrained engineering benchmarks. The tension/compression spring design, the pressure vessel design, the welded beam design, the speed reducer design, the gear train design, and the three-bar truss design are all part of the engineering design problems. The FROBLAO runs independently for each engineering problem, with a selected Aquila population size of 30, with 500 iterations, and the number of function evaluations (NFEs) of 15,000.

### 4.1 Tension/compression spring design problem

**Figure 11:**
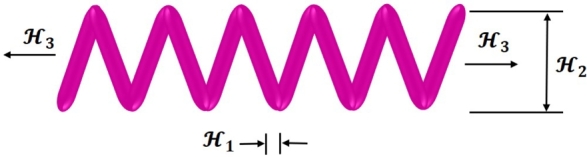
The design of the tension/compression spring problem.

The engineering design problem of a tension/compression spring problem is designated in [90]. The primary goal of this problem is to reduce the weight of a given spring. The design of the spring problem is shown in Fig. 11. Three choice factors were used in this design problem: the diameter of the wire ($H_{1}$), the average coil diameter ($H_{2}$), and the overall amount of active coils ($H_{3}$).

This design problem can be expressed mathematically as follows:

$$\text{Consider}\;\overset{\to }{H}=[H_{1}\;H_{2}\;H_{3}],\text{Minimize}\;f(\overset{\to }{H})=(H_{3}+2)H_{2}H_{1}^{2},\text{Subject to}\;g_{1}\;(\overset{\to }{H})=1-\frac{H_{2}^{3}H_{3}}{71785H_{1}^{4}}\leq 0,g_{2}\;(\overset{\to }{H})=\frac{4H_{2}^{2}-H_{1}H_{2}}{12566(H_{2}H_{1}^{3}-H_{1}^{4})}+\frac{1}{5108H_{1}^{2}}\leq 0,g_{3}\;(\overset{\to }{H})=1-\frac{140.45H_{1}}{H_{2}^{2}H_{3}}\leq 0,g_{4}\;(\overset{\to }{H})=\frac{H_{1}+H_{2}}{1.5}-1\leq 0,\text{Variable range}\;0.05\leq H_{1}\leq 2.00,0.25\leq H_{2}\leq 1.30,2.00\leq H_{3}\leq 15.0.$$

**Figure 12:**
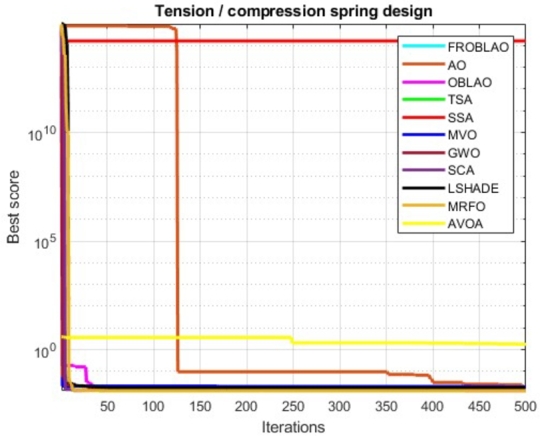
Convergence graphs for the tension/compression spring problem.

Table 18 compares the performance of FROBLAO with standard algorithms. The outcomes demonstrate that FROBLAO has provided the solution to this problem with optimal values for variables (5.993E-02, 5.850E-01, and 4.651E+00) and an optimal solution of 1.276E-02. Table 18 shows that FROBLAO outperformed other algorithms. The convergence graphs for FROBLAO's superior results and those of the other algorithms under investigation for comparison are shown in Fig. 12.

**Table 18:** The tension/compression spring problem's comparing results.

Algorithms	Optimum values for factors			Optimum weight
	$H_{1}$	$H_{2}$	$H_{3}$	
FROBLAO	5.993E-02	5.850E-01	4.651E+00	1.276E-02
AO	6.189E-02	5.704E-01	6.212E+00	1.794E-02
OBLAO	6.551E-02	7.750E-01	2.956E+00	1.648E-02
TSA	5.000E-02	3.169E-01	1.411E+01	1.276E-02
SSA	6.871E-02	1.334E+00	1.177E+00	1.627E+14
MVO	6.989E-02	9.727E-01	2.000E+00	1.900E-02
GWO	5.355E-02	4.030E-01	9.045E+00	1.398E-02
SCA	5.134E-02	3.468E-01	1.203E+01	1.283E-02
LSHADE	6.899E-02	9.334E-01	2.000E+00	1.777E-02
MRFO	5.398E-02	4.144E-01	8.575E+00	1.277E-02
AVOA	5.534E-02	4.511E-01	7.335E+00	1.290E-02

### 4.2 Pressure vessel design problem

**Figure 13:**
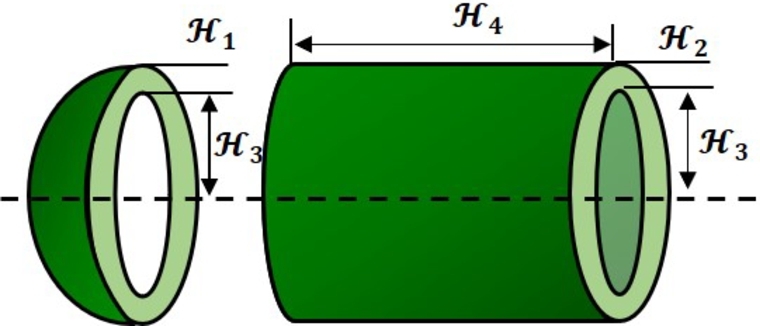
The design of the pressure vessel problem.

The idea is to produce a pressure vessel design with the least cost. Fig. 13 illustrates the pressure vessel and the design parameters. This problem has four variables: shell thickness ($H_{1}$), head thickness ($H_{2}$), inner radius ($H_{3}$), and length of the cylindrical section excluding the head ($H_{4}$).

This design problem can be expressed mathematically as follows:

$$\text{Consider}\;\overset{\to }{H}=[H_{1}\;H_{2}\;H_{3}\;H_{4}],\text{Minimize}\;f(\overset{\to }{H})=0.6224H_{1}H_{3}H_{4}+1.7781H_{2}H_{3}^{2}+3.1661H_{1}^{2}H_{4}+19.84H_{1}^{2}H_{3},\text{Subject to}\;g_{1}\;(\overset{\to }{H})=-H_{1}+0.0193H_{3}\leq 0,g_{2}\;(\overset{\to }{H})=-H_{1}+0.00954H_{3}\leq 0,g_{3}\;(\overset{\to }{H})=-\pi H_{3}^{2}H_{4}-\frac{4}{3}\pi H_{3}^{2}+1,296,000\leq 0,g_{4}\;(\overset{\to }{H})=H_{4}-240\leq 0,\text{Variable range}\;0\leq H_{1}\leq 99,0\leq H_{2}\leq 99,10\leq H_{3}\leq 200,10\leq H_{4}\leq 20.$$

**Figure 14:**
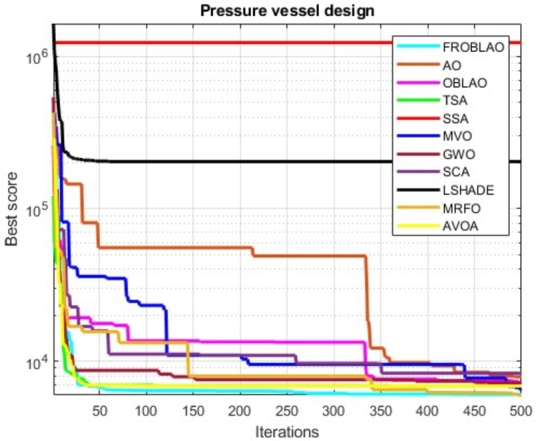
Convergence graphs for the pressure vessel problem.

Table 19 compares the performance of FROBLAO with standard algorithms. The outcomes demonstrate that FROBLAO has provided the solution to this problem with optimal values for variables (7.842E-01, 3.891E-01, 4.058E+01, and 1.995E+02) and an optimal solution of 5.975E+03. Table 19 shows that FROBLAO outperformed other algorithms. The convergence graphs for FROBLAO's superior results and those of the other algorithms under investigation for comparison are shown in Fig. 14.

**Table 19:** The pressure vessel problem's comparative results.

Algorithms	Optimum values for factors				Optimum weight
	$H_{1}$	$H_{2}$	$H_{3}$	$H_{4}$	
FROBLAO	7.842E-01	3.891E-01	4.058E+01	1.995E+02	5.975E+03
AO	1.292E+00	6.636E-01	6.579E+01	1.023E+01	7.882E+03
OBLAO	1.243E+00	6.035E-01	6.322E+01	1.894E+01	7.247E+03
TSA	1.115E+00	5.559E-01	5.711E+01	5.102E+01	6.857E+03
SSA	2.596E+01	2.304E+01	6.331E+01	6.936E+01	1.230E+06
MVO	9.922E-01	4.928E-01	5.112E+01	8.985E+01	6.405E+03
GWO	1.221E+00	6.039E-01	6.323E+01	1.901E+01	7.173E+03
SCA	1.236E+00	5.541E-01	5.683E+01	7.023E+01	8.316E+03
LSHADE	1.000E+01	1.000E+01	5.368E+01	7.159E+01	2.043E+05
MRFO	7.937E-01	4.334E-01	4.109E+01	1.896E+02	6.041E+03
AVOA	1.147E+00	5.669E-01	5.943E+01	3.759E+01	6.775E+03

### 4.3 Welded beam design problem

**Figure 15:**
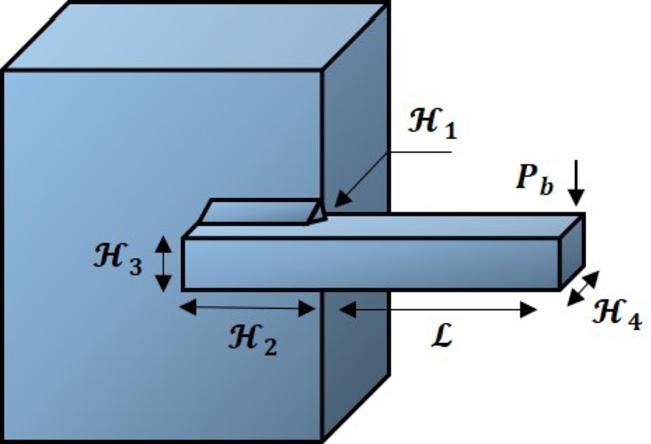
The design of the welded beam design problem.

A popular welded beam design [91] is given in Fig. 15 to examine the demonstration of FROBLAO in the engineering area. The goal is to discover the optimal design factors for reducing the total manufacturing cost of a welded beam. This problem has seven constraints and four variables: weld thickness ($H_{1}$), bar length ($H_{2}$), height ($H_{3}$), and thickness ($H_{4}$).

This design problem can be expressed mathematically as follows:

$$\text{Consider}\;\overset{\to }{H}=[H_{1}\;H_{2}\;H_{3}\;H_{4}],\text{Minimize}\;f(\overset{\to }{H})=1.10471H_{1}^{2}H_{2}+0.04811H_{3}H_{4}(14.0+H_{2}),\text{Subject to}\;g_{1}\;(\overset{\to }{H})=\tau (\overset{\to }{H})-\tau_{max}\leq 0,g_{2}\;(\overset{\to }{H})=\sigma (\overset{\to }{H})-\sigma_{max}\leq 0,g_{3}\;(\overset{\to }{H})=\delta (\overset{\to }{H})-\delta_{max}\leq 0,g_{4}\;(\overset{\to }{H})=H_{1}-H_{4}\leq 0,g_{5}\;(\overset{\to }{H})=P-P_{c}(\overset{\to }{H})\leq 0,g_{6}\;(\overset{\to }{H})=0.125-H_{1}\leq 0,g_{7}\;(\overset{\to }{H})=1.1047H_{1}^{2}-0.04811H_{3}H_{4}(14.0+H_{2})-5.0\leq 0,\text{Variable range}\;0.1\leq H_{1}\leq 2,0.1\leq H_{2}\leq 10,0.1\leq H_{3}\leq 10,0.1\leq H_{4}\leq 2,\text{where}\;\tau (\overset{\to }{H})=\sqrt{{\left(\tau^{\prime }\right)}^{2}+2\tau^{\prime }\tau^{\prime \prime }\frac{H_{2}}{2R}+{\left(\tau^{\prime \prime }\right)}^{2}},\tau^{\prime }=\frac{P}{\sqrt{2}H_{1}H_{2}},\tau^{\prime \prime }=\frac{MR}{J},M=P(L+\frac{H_{2}}{2}),R=\sqrt{\frac{H_{2}^{2}}{4}+{\left(\frac{H_{1}+H_{3}}{2}\right)}^{2}},$$

$$J=2\{\sqrt{2}H_{1}H_{2}[\frac{H_{2}^{2}}{4}+{\left(\frac{H_{1}+H_{3}}{2}\right)}^{2}]\},\sigma (\overset{\to }{H})=\frac{6PL}{H_{4}H_{3}^{2}},\delta (\overset{\to }{H})=\frac{6PL^{3}}{EH_{3}^{2}H_{4}},P_{c}(\overset{\to }{H})=\frac{4.013E\sqrt{\frac{H_{3}^{2}H_{4}^{6}}{36}}}{L^{2}}(1-\frac{H_{3}}{2L}\sqrt{\frac{E}{4G}}),P=6.00E+03\;lb,L=14\;in,\delta_{max}=0.25\;in,E=3.00E+07\;psi,G=1.20E+07\;psi,\tau_{max}=1.36E+04\;psi,\sigma_{max}=1.36E+04\;psi.$$

**Figure 16:**
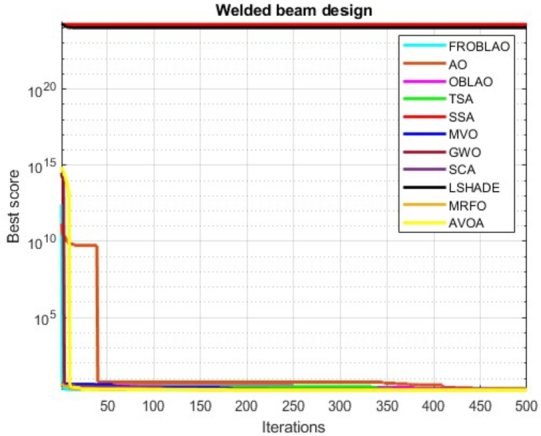
Convergence graphs for the welded beam problem.

Table 20 compares the performance of FROBLAO with standard algorithms. The outcomes demonstrate that FROBLAO has provided the solution to this problem with optimal values for variables (1.986E-01, 3.687E+00, 9.067E+00, and 2.056E-01) and an optimal solution of 1.725E+00. Table 20 shows that FROBLAO outperformed other algorithms. The convergence graphs for FROBLAO's superior results and those of the other algorithms under investigation for comparison are shown in Fig. 16.

**Table 20:** The welded beam problem's comparative results.

Algorithms	Optimum values for factors				Optimum weight
	$H_{1}$	$H_{2}$	$H_{3}$	$H_{4}$	
FROBLAO	1.986E-01	3.687E+00	9.067E+00	2.056E-01	1.725E+00
AO	2.478E-01	2.895E+00	9.249E+00	2.591E-01	2.145E+00
OBLAO	1.746E-01	4.320E+00	9.101E+00	2.073E-01	1.809E+00
TSA	1.955E-01	3.775E+00	9.117E+00	2.058E-01	1.764E+00
SSA	1.398E+00	9.842E-01	1.949E+00	1.848E+00	1.750E+24
MVO	1.901E-01	3.714E+00	9.400E+00	2.040E-01	1.783E+00
GWO	1.976E-01	3.667E+00	9.030E+00	2.060E-01	1.740E+00
SCA	1.775E-01	3.994E+00	9.866E+00	2.034E-01	1.876E+00
LSHADE	1.963E+00	1.939E+00	2.000E+00	2.000E+00	1.089E+24
MRFO	2.056E-01	3.469E+00	9.036E+00	2.056E-01	1.747E+00
AVOA	1.742E-01	4.308E+00	9.038E+00	2.057E-01	1.782E+00

### 4.4 Speed reducer design problem

**Figure 17:**
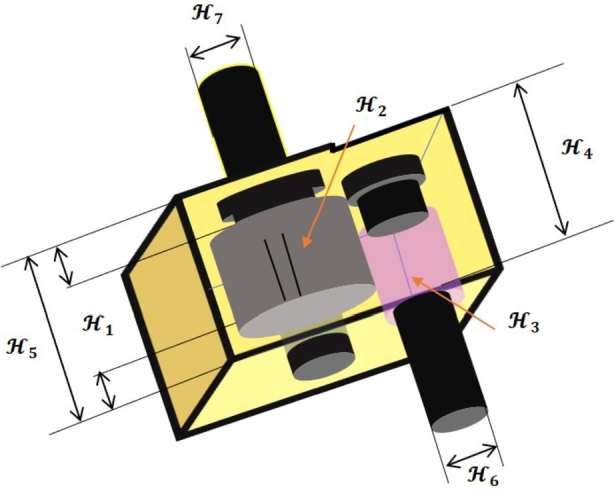
The design of the speed reducer design problem.

The speed reducer [92], is a crucial component of the gearbox in mechanical systems and has a wide range of uses. In this optimization problem, the weight of the speed reducer has to be lowered with 11 constraints (see Fig. 17). Seven variables make up this problem, such as $H_{1}$, $H_{2}$, $H_{3}$, $H_{4}$, $H_{5}$, $H_{6}$, and $H_{7}$.

This design problem can be expressed mathematically as follows:

$$\text{Consider}\;\overset{\to }{H}=[H_{1}\;H_{2}\;H_{3}\;H_{4}\;H_{5}\;H_{6}\;H_{7}],\text{Minimize}\;f(\overset{\to }{H})=0.7854H_{1}H_{2}^{2}(3.3333H_{3}^{2}+14.9334H_{3}-43.0934)-1.508H_{1}(H_{6}^{2}+H_{7}^{2})+7.4777(H_{6}^{2}+H_{7}^{2})+0.7854(H_{4}H_{6}^{2}+H_{5}H_{7}^{2}),\text{Subject to}\;g_{1}\;(\overset{\to }{H})=\frac{27}{H_{1}H_{2}^{2}H_{3}}-1\leq 0,g_{2}\;(\overset{\to }{H})=\frac{397.5}{H_{1}H_{2}^{2}H_{3}^{2}}-1\leq 0,g_{3}\;(\overset{\to }{H})=\frac{1.93H_{4}^{3}}{H_{2}H_{6}^{4}H_{3}}-1\leq 0,g_{4}\;(\overset{\to }{H})=\frac{1.93H_{5}^{3}}{H_{2}H_{7}^{4}H_{3}}-1\leq 0,g_{5}\;(\overset{\to }{H})=\frac{\sqrt{{\left(\frac{745H_{4}}{H_{2}H_{3}}\right)}^{2}+16.9\times 10^{6}}}{110H_{6}^{3}}-1\leq 0,g_{6}\;(\overset{\to }{H})=\frac{\sqrt{{\left(\frac{745H_{5}}{H_{2}H_{3}}\right)}^{2}+157.5\times 10^{6}}}{85H_{7}^{3}}-1\leq 0,g_{7}\;(\overset{\to }{H})=\frac{H_{2}H_{3}}{40}-1\leq 0,g_{8}\;(\overset{\to }{H})=\frac{5H_{2}}{H_{1}}-1\leq 0,g_{9}\;(\overset{\to }{H})=\frac{H_{1}}{12H_{2}}-1\leq 0,g_{10}\;(\overset{\to }{H})=\frac{1.5H_{6}+1.9}{H_{4}}-1\leq 0,g_{11}\;(\overset{\to }{H})=\frac{1.1H_{7}+1.9}{H_{5}}-1\leq 0,$$

$$\text{Variable range}\;2.6\leq H_{1}\leq 3.6,0.7\leq H_{2}\leq 0.8,H_{3}\in \{17,18,19,....,28\},7.3\leq H_{4},H_{5}\leq 8.3,2.9\leq H_{6}\leq 3.9,5\leq H_{7}\leq 5.5.$$

**Figure 18:**
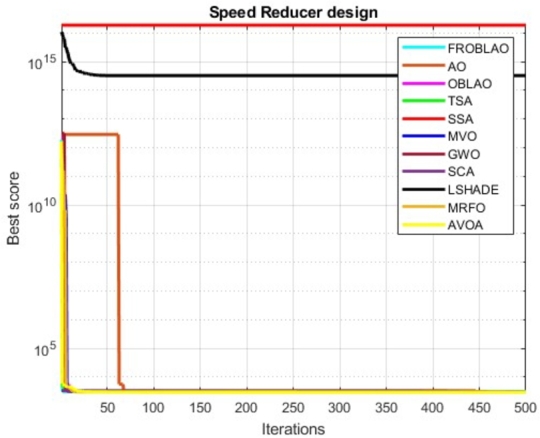
Convergence graphs for the speed reducer problem.

Table 21 compares the performance of FROBLAO with standard algorithms. The outcomes demonstrate that FROBLAO has provided the solution to this problem with optimal values for variables (3.536E+00, 7.000E-01, 1.700E+01, 7.380E+00, 7.885E+00, 3.355E+00, and 5.291E+00) and an optimal solution of 2.994E+03. Table 21 shows that FROBLAO outperformed other algorithms. The convergence graphs for FROBLAO's superior results and those of the other algorithms under investigation for comparison are shown in Fig. 18.

**Table 21:** The speed reducer design problem's comparative results.

Algorithms	Optimum values for factors							Optimum weight
	$H_{1}$	$H_{2}$	$H_{3}$	$H_{4}$	$H_{5}$	$H_{6}$	$H_{7}$	
FROBLAO	3.5359	0.7	17	7.3803	7.8849	3.3549	5.2906	2994.4739
AO	3.6	0.7	17	8.3	7.9518	3.3999	5.3037	3071.7139
OBLAO	3.5476	0.7	17.0455	8.2744	7.7850	3.4791	5.2924	3069.7601
TSA	3.5817	0.7	17	7.3	8.3	3.3655	5.2869	3043.4951
SSA	3.5134	2.7941	3.1298	3.5494	2.8633	3.0239	3.3793	1.878E+16
MVO	3.5106	0.7	17	7.5247	8.1840	3.4735	5.2869	3043.8232
GWO	3.5046	0.7	17	7.4628	8.0724	3.4021	5.2909	3021.8463
SCA	3.6	0.7	17	7.3	8.3	3.4702	5.3891	3144.8691
LSHADE	5.5	1.4308	5.5	5.5	5.5	3.2804	5.1030	3.270E+14
MRFO	3.5	0.7	17	7.3	7.7153	3.3502	5.2866	3016.7605
AVOA	3.5	0.7	17	8.0272	8.1225	3.3516	5.2867	3010.2794

### 4.5 Gear train design problem

**Figure 19:**
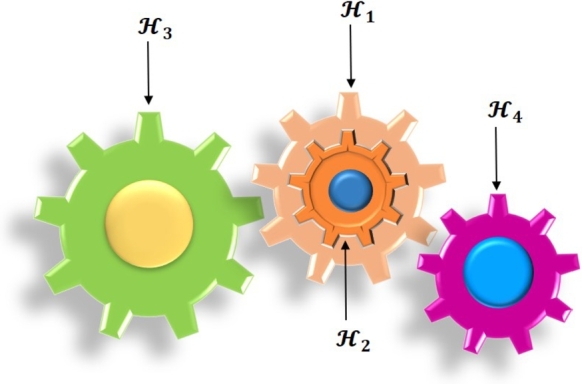
The design of the gear train problem.

Sandgren proposed the design of a gear train [93]. This problem aims to reduce the gear ratio, which is the ratio of angular velocities between input and output shafts. In this problem, the variables that represent the overall amount of teeth in the gears are $A(H_{1})$, $B(H_{2})$, $C(H_{3})$, and $D(H_{4})$. Fig. 19 depicts the gear train problem.

This design problem can be expressed mathematically as follows:

$$\text{Consider}\;\overset{\to }{H}=[H_{1}\;H_{2}\;H_{3}\;H_{4}],\text{Minimize}\;f(\overset{\to }{H})={\left(\frac{1}{6.931}-\frac{H_{3}H_{2}}{H_{1}H_{4}}\right)}^{2},\text{Variable range}\;H_{1}\;H_{2}\;H_{3}\;H_{4}\in \{12,13,14,\cdots ,60\}.$$

**Figure 20:**
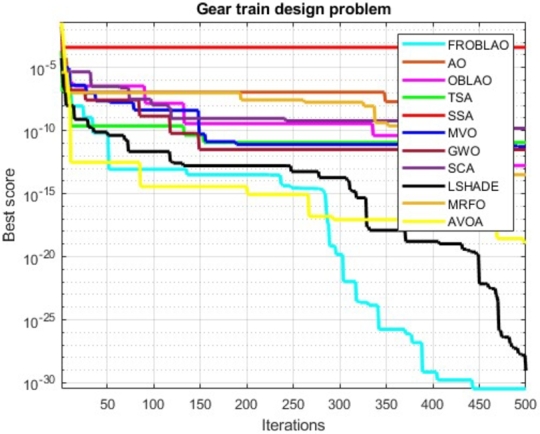
Convergence graphs for the gear train problem.

Table 22 compares the performance of FROBLAO with standard algorithms. The outcomes demonstrate that FROBLAO has provided the solution to this problem with optimal values for variables (5.918E+01, 1.640E+01, 3.123E+01, and 5.999E+01) and an optimal solution of 1.233E-32. Table 22 shows that FROBLAO outperformed other algorithms. The convergence graphs for FROBLAO's superior results and those of the other algorithms under investigation for comparison are shown in Fig. 20.

**Table 22:** The gear train problem's comparative results.

Algorithms	Optimum values for factors				Optimum weight
	$H_{1}$	$H_{2}$	$H_{3}$	$H_{4}$	
FROBLAO	5.918E+01	1.640E+01	3.123E+01	5.999E+01	1.233E-32
AO	5.246E+01	3.049E+01	1.200E+01	4.834E+01	3.058E-12
OBLAO	5.093E+01	3.359E+01	1.261E+01	5.767E+01	1.743E-13
TSA	6.000E+01	2.096E+01	1.504E+01	3.642E+01	1.160E-11
SSA	4.660E+01	2.238E+01	1.751E+01	5.123E+01	3.968E-04
MVO	4.526E+01	3.086E+01	1.200E+01	5.672E+01	5.224E-12
GWO	6.001E+01	2.415E+01	1.201E+01	3.348E+01	3.182E-12
SCA	4.394E+01	1.937E+01	1.478E+01	4.519E+01	1.537E-10
LSHADE	5.945E+01	2.091E+01	2.390E+01	5.827E+01	3.397E-31
MRFO	5.998E+01	4.324E+01	1.200E+01	5.998E+01	1.374E-19
AVOA	5.447E+01	1.356E+01	1.948E+01	3.363E+01	9.322E-30

### 4.6 Three-bar truss design problem

**Figure 21:**
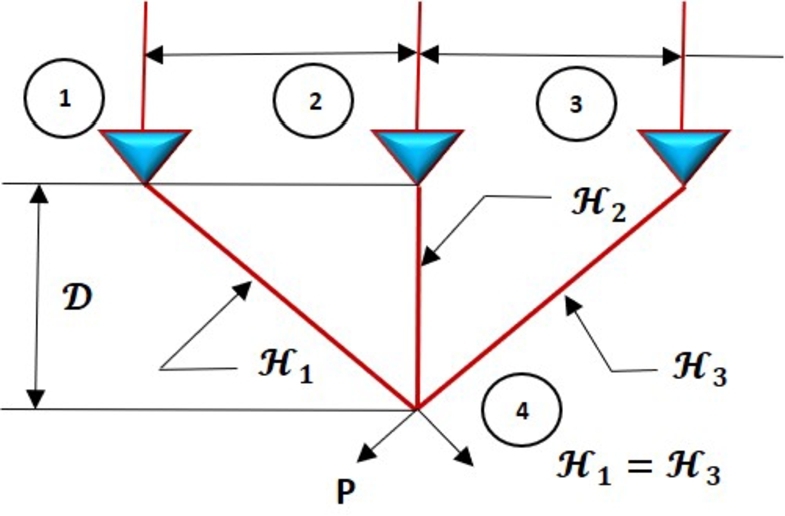
The design of the three-bar truss problem.

In this case, a three-bar planar truss is depicted in Fig. 21. While stress (*σ*) limits on each truss member are maintained, the volume of a statically loaded 3-bar truss must be reduced. The aim is to find the best cross-sectional areas, $H_{1}$ and $H_{2}$.

This design problem can be expressed mathematically as follows:

$$\text{Consider}\;\overset{\to }{H}=[H_{1}\;H_{2}],\text{Minimize}\;f(\overset{\to }{H})=(2\sqrt{2H_{1}}+H_{2})\times l,\text{Subject to}\;g_{1}\;(\overset{\to }{H})=\frac{\sqrt{2H_{1}}+H_{2}}{\sqrt{2H_{1}^{2}}+2H_{1}H_{2}}P-\sigma \leq 0,g_{2}\;(\overset{\to }{H})=\frac{H_{2}}{\sqrt{2H_{1}^{2}}+2H_{1}H_{2}}P-\sigma \leq 0,g_{3}\;(\overset{\to }{H})=\frac{1}{\sqrt{2H_{2}}+H_{1}}P-\sigma \leq 0,l=100cm,P=2kN/{\left(cm\right)}^{3},\sigma =2kN/{\left(cm\right)}^{3},\text{Variable range}\;0\leq H_{1},H_{1}\leq 1.$$

**Figure 22:**
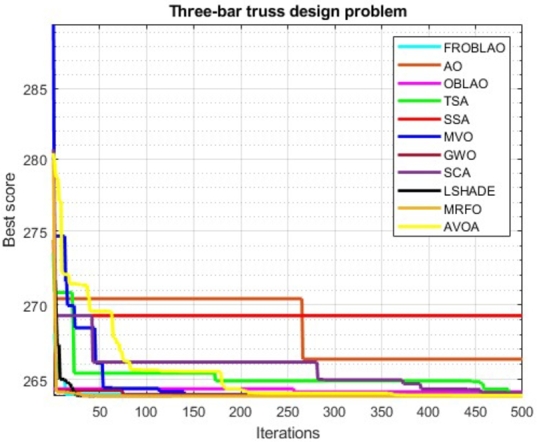
Convergence graphs for the three-bar truss problem.

Table 23 compares the performance of FROBLAO with standard algorithms. The outcomes demonstrate that FROBLAO has provided the solution to this problem with optimal values for variables (7.880E-01 and 4.113E-01) and an optimal solution of 264.0251. Table 23 shows that FROBLAO outperformed other algorithms. The convergence graphs for FROBLAO's superior results and those of the other algorithms under investigation for comparison are shown in Fig. 22.

**Table 23:** The three-bar truss problem's comparative results.

Algorithms	Optimum values for factors		Optimum weight
	*H*_1_	*H*_2_	
FROBLAO	7.880E-01	4.113E-01	264.0251
AO	7.610E-01	5.111E-01	266.3447
OBLAO	7.915E-01	4.030E-01	264.1778
TSA	7.881E-01	4.103E-01	263.9257
SSA	8.721E-01	2.255E-01	269.2571
MVO	7.924E-01	3.979E-01	263.9087
GWO	7.877E-01	4.109E-01	263.9106
SCA	7.729E-01	4.554E-01	264.1940
LSHADE	7.887E-01	4.082E-01	263.8958
MRFO	7.885E-01	4.089E-01	263.8958
AVOA	7.961E-01	3.876E-01	263.9354

## 5 Conclusion

This paper introduced an improved Aquila optimizer by employing the Fast Random Opposition-Based Learning strategy (FROBL), and the new strategy is named FROBLAO. This strategy targets enhancing the convergence speed and escapes from local optima by generating a new opposite solution to the current solution. The FROBLAO is tested using CEC 2005, CEC2019, and CEC 2020 test functions, and the results are compared with eleven competitive algorithms. The experimental results demonstrate that the suggested FROBLAO improves optimization by maintaining stability throughout exploration, exploitation, and quicker convergence. In addition, the FROBLAO is employed with six real-life engineering problems, and the results were compared with other state-of-the-art meta-heuristic algorithms. The statistical outcomes show that the FROBLAO also performs superior in engineering optimization problems compared to other meta-heuristic techniques. In future work, we will be able to apply FROBLAO to feature selection, combinatorial optimization problems, job scheduling, and stress suitability. Future versions may include binary and multi-objective features.

## CRediT authorship contribution statement

**S. Gopi:** Writing – original draft, Validation, Methodology, Formal analysis, Data curation. **Prabhujit Mohapatra:** Writing – review & editing, Validation, Supervision, Resources, Methodology, Investigation, Conceptualization.

## Declaration of Competing Interest

The authors declare that they have no known competing financial interests or personal relationships that could have appeared to influence the work reported in this paper.

## Data Availability

No data was used for the research described in the article.
